# HELP-TCR: Harmonized Explainable Language Processing Toolkit for T Cell Antigen Receptor Repertoires

**DOI:** 10.34133/csbj.0117

**Published:** 2026-08-21

**Authors:** Yulyana Kalesnik, Dawid Krawczyk, Maciej Pietrzak, Michał Seweryn

**Affiliations:** ^1^ Centre for Digital Biology and Biomedical Sciences, University of Lodz, Lodz, Poland.; ^2^ Regional Digital Medicine Center, Copernicus Memorial Hospital and University of Lodz, Lodz, Poland.; ^3^ University of Lodz Doctoral School of Exact and Natural Sciences, University of Lodz, Lodz, Poland.; ^4^ Department of Biomedical Informatics, The Ohio State University, Columbus, OH, USA.; ^5^ College of Medicine, The Ohio State University, Columbus, OH, USA.

## Abstract

Functional characterization of T cell antigen receptor (TCR) repertoires is critical for understanding adaptive immune responses across diverse contexts, including infectious diseases, cancer, autoimmune conditions, and allergic disorders. Detailed analysis of TCR repertoires can reveal disease-specific signatures and support biomarker discovery and the development of immunotherapies as well as vaccines. Current computational approaches often prioritize global repertoire metrics or employ deep learning models that, while powerful, offer limited interpretability. Here, we present HELP-TCR, a novel machine learning framework based on natural language processing that integrates low-dimensional, explainable feature extraction with robust sample classification performance. HELP-TCR aims to classify the per-sample TCR repertoires via a nonparametric probabilistic approach. TCR repertoires are represented by modeling within sample position-specific distributions of single amino acids and amino acid pairs, transforming sequences into multidimensional tensor structures. To increase reproducibility, a consensus grouping method is proposed to merge the features with highly similar position-wise distributions. A modified ResNet-18 deep learning architecture, adapted to process these tensors, enables accurate sample classification, while post hoc analysis based on saliency map highlights the most informative features contributing to model predictions. Using a dataset of bootstrapped TCR sequences, HELP-TCR achieved an area under the curve (AUC) of 0.96, outperforming existing methods including DeepTCR (AUC 0.76) and TCR-BERT embeddings, which exhibited limited class separability. Beyond performance, HELP-TCR enables identification of position-specific amino acid motifs (pairs) associated with sample TCR repertoire classification decisions, offering biologically interpretable insights into TCR repertoire differences. By emphasizing model interpretability alongside predictive accuracy, HELP-TCR provides a versatile platform for functional TCR repertoire analysis with potential applications in immunotherapy development, vaccine design, and immune monitoring.

## Introduction

The adaptive immune system depends on the diverse repertoire of T cell receptors (TCRs) to detect a broad range of antigens, including those derived from pathogens and malignant cells [1]. High-throughput sequencing of TCR repertoires has become an important tool in immunology, supporting studies of clonal expansion, diversity, and repertoire overlap across various biological contexts [2]. Traditional analytical approaches typically focus on global characteristics of TCR repertoires, such as diversity indices or the prevalence of dominant clones, but these methods provide limited insight into sequence-specific features associated with antigen recognition [3]. In recent years, deep learning models inspired by natural language processing (NLP) have been applied to TCR sequence data to address this limitation.

In this study, we present a novel method, HELP-TCR (together with a user-friendly implementation in Python), designed to bridge the gap between classical tools—which focus primarily on diversity, overlap, and clonal dynamics—and novel deep learning methods based on high-dimensional embeddings. While the latter methods differ from classical tools, they often act as “black boxes”, failing to provide sufficient information regarding specific molecular features (i.e., physicochemical properties) that drive classification tasks. We evaluate the performance of our model against existing deep learning algorithms for TCR analyses, which include both per-sample classification (DeepTCR) and embeddings based on antigen specificity grouping [TCR-BERT (bidirectional encoder representations from transformers)]. However, our primary objective is not only to compare performance metrics but also to analyze the algorithmic differences, highlighting how our approach enables feature engineering in the context of per-sample classification. Here, we stress that our representation of data is suitable for per-sample classification of TCR repertoires where the footprint of divergence between groups of samples may be seen as a result of moderate TCR-pMHC (peptide–major histocompatibility complex) differential interactions driven by differential abundance of antigens with high similarity to self-antigens as in the comparison between tumor microenvironment and whole blood or comparisons between tissues.

In HELP-TCR, we perform a comprehensive analysis of amino acid composition and motifs within TCR sequences, using appropriate indices/metrics to quantify the differences between the underlying distributions of features across samples. These indices allow us to perform overlap analysis between TCR populations and select informative features with the aid of standard supervised and unsupervised machine learning tools. We focused on features defined as single amino acids or amino acid pairs, offering a low-dimensional alternative to autoencoder-based methods that rely on complex, high dimensional motif representations. This design choice mitigates the likelihood of overfitting and enhances model interpretability.

Furthermore, we introduce a simple representation of the TCR repertoire as a tensor, enabling the direct application of deep learning methods for per-sample classification. Under this simple model with no high dimensional motifs and non-informative representations performed with the aid of embeddings, we achieve a relatively high level of confidence in discriminating between the classes of samples. Building on the representation and analysis of low dimensional motifs, we provide a set of tools for evaluation of position-specific patterns of amino acids in the context of discrimination.

We compare our approach with other deep learning algorithms, which were developed to analyze TCR repertoires. The first one is based on a well-known BERT architecture [4]. TCR-BERT [5] is a deep learning method designed to analyze TCR repertoires and predict TCR binding interactions using a transformer-based NLP models. The algorithm is structured to process amino acid sequences of TCRs as well as their corresponding pMHC interactions. By leveraging the self-attention mechanism of BERT, TCR-BERT learns meaningful representations of TCR sequences, capturing underlying biochemical and structural patterns. Here, one may allow for subword tokenization, where tokens correspond to amino acid subunits rather than individual residues. The architecture includes not only tokenization of TCR sequences but also embedding layers to encode sequence features, multiple layers to extract contextual dependencies, and output layers for classification.

At the same time, the DeepTCR algorithm [6] is based on the variational autoencoder architecture (as briefly described in subsequent sections). This approach was primarily designed for descriptive/exploratory analyses of TCR motifs as well as building predictive models based on high-dimensional embeddings. In particular, in the unsupervised setting, DeepTCR may be used to group TCR sequences according to the similarity of the corresponding motif distributions. At the same time, the autoencoder-based embedding enables building predictive models for classification and identification of group-specific motifs.

### Language processing for TCR repertoire analysis

We propose a set of methods that consistently focus on the composition of amino acids, or pairs of amino acids, and their position-wise distribution, to enable comprehensive comparison of TCR repertoires. This approach is an alternative to the standard comparison of frequencies of particular receptors and classical models for motif discovery [7]. In the standard setting, the comparisons may be heavily biased by the fraction of unobserved features, which is likely to differ between samples. At the same time, many models have been utilized to find common combinations of amino acids that may serve as signatures of antigen response. In what follows, we highlight the unique features of our approach over the discovery of common motifs.

Many models seek common amino acid combinations as signatures of antigen response, but we adopt a different strategy. We argue that redundancy in TCR–antigen interactions is a cornerstone of the repertoire in both health and disease [8]. While immune responses to foreign antigens typically decrease diversity and increase clonality, diversity itself is a key characteristic of the “healthy” repertoire [9]. However, this diversity is constrained by negative thymic selection, which eliminates TCRs with high affinity for self-antigens.

These factors—high diversity and weak interactions with self-antigens [10]—suggest that the assumption of existence of long motifs is only valid when considering strong interactions with foreign antigens (the “key-lock” model). Therefore, focusing solely on large motifs that discriminate between repertoires can lead to false positives, particularly in deep learning contexts where the risk of overfitting is higher than in explainable machine learning.

Conceptually, we posit that to avoid overfitting, one should identify multiple combinations of smaller motifs and cluster them into subsets. These subsets likely carry redundant information regarding the antigen repertoire shaping the TCRs—which is to us more natural and reflects the inherent diversity of the TCR repertoire. This parallels regularized machine learning models, where multiple weak associations are often preferred over a few strong predictors. Crucially, models built on multiple predictors carrying redundant information require methods that ensure explainability. To this end, we implement saliency maps [11].

We emphasize that we do not claim that our approach is superior to large language model (LLM)-based methods for identifying large motifs associated with foreign antigen responses. Instead, we re-consider and examine in detail the assumption that strong, common predictors are the only valid metric for TCR repertoire comparison. Our method supports the development of broader analytical tools suitable for comparisons beyond the context of strong immune responses.

Since the TCR β chain consists of low-diversity regions at the ends, in our analyses, we propose to use a position-wise dependence for the motifs. Due to high complexity, we only select k=1 or k=2 for position-wise dependence. For k=1, the above-described model is obvious and results in a matrix with rows corresponding to the position on the peptide, whereas the columns represent the elements of the alphabet. For pairs of amino acids, we propose 2 different approaches. One is based on a sliding window, and therefore also results in a matrix representation where the rows correspond to the position of the first amino acid and the columns correspond to the pairs of amino acids. Our second approach is not based on a sliding window, but on the selection of any 2 amino acids in the peptide with all information on the position of both amino acids kept in the resulting data structure. This leads to a tensor representation of the peptide, which we describe in more detail in the section below. We provide a schematic diagram of our approach in Fig. S7.

## Materials and Methods

### Dataset

In this paper, we use a dataset generated as part of the study [12]. For more details, please refer to the source paper on the details of the data generation and sample collection. Here, we recall certain quality control measures that have been taken in the original study to make the reader aware of the data transformation.

The authors of [12] aimed to ensure reliable T cell receptor (TCR) identification; thus, several filtering criteria were applied. First, a minimum total read count threshold of 5 was set to exclude low-abundance rearrangements, which helped reduce sequencing noise. Additionally, only productive TCR rearrangements were retained by setting the parameter productiveOnly = True, thereby excluding nonproductive recombinations. TCRs were analyzed based on their amino acid sequences rather than nucleotide sequences, which enhanced functional comparability. Furthermore, TCR sequences likely to be generated by polymerase chain reaction (PCR) amplification errors were removed to avoid artificial inflation of diversity. In all our further analyses, we remove the smallest class of samples to simplify our reasoning (i.e., avoid multiclass comparisons) and allow for estimation of robustness of predictions by means of bootstrap analyses.

The analyses performed originally on the dataset of interest included standard metrics, which quantify the diversity of individual TCR repertoires as well as estimation of the degree of overlap between all samples. Briefly, the number of unique T cell rearrangements was quantified as the richness estimator of the individual TCR repertoire. To ensure robustness and fairness of richness comparisons, extrapolation was performed to achieve 400,000 templates for peripheral blood mononuclear cells (PBMCs) and 120,000 templates for tissue to normalize for sampling depth. Beyond richness, clonality was evaluated, as defined by 1 − Peilou’s evenness, and clustering of TCRs into similar groups was performed by means of the GLIPH algorithm [13].

### Data preparation for DeepTCR and TCR-BERT

To ensure a fair comparison (between HELP-TCR, DeepTCR, and TCR-BERT) and evaluate the variability/stability of the results, 3 distinct bootstrap regimens were employed for classification algorithms: (a) when the replicate from the same sample is present in both the training and testing cohort, (b) when the model is trained only on mixed samples (2 original samples are mixed as one) and testing is done on bootstrap replicates of the original samples, (c) a group-wise split, where the original samples are first divided into the training and testing and subsequently bootstrap replicates are performed.

For TCR-BERT, we utilized 89 samples, with each sample containing 1,000 bootstrapped sequences. Transformer embedding was applied to each sequence within the sample, resulting in a data structure with dimensions 89,000 × 768 (i.e., the number of TCRs by the dimensionality of the latent space) as a result of the TCR-BERT procedure. To increase the discriminative power and visualize the results in the latent space, we utilized the Uniform Manifold Approximation and Projection (UMAP) algorithm to embed the results in a 2-dimensional (2D) space. Following this, we applied the Leiden clustering algorithm in 2 dimensions to assess the separation of clusters. Upon application of the Leiden algorithm, we identified the 10 large clusters (and numerous small clusters containing less structured observations) and proceeded with further analysis based on the set of 10 largest clusters. In this analysis, we evaluated the separation of clusters with the aid of the Wasserstein distance [14], computed the fraction of N and T samples per cluster, and performed per-cluster visualization by means of logo plots.

For the DeepTCR algorithm, we used the same bootstrapped samples as in the TCR-BERT implementation. However, for DeepTCR, one-hot encoding was applied to each sequence in the sample before the embedding layer. Subsequently, we trained the DeepTCR algorithm in a loop with different data splits to assess the variability in performance of the model instead of obtaining one point estimate of performance.

### Data-driven approach of filtering data for per-sample classification

Before testing any of the modules presented in this study, it is essential to describe the standardization procedures applied to the samples and sequences within the TCR repertoire. To achieve this, we first developed a ranking system that selects the most optimal peptide length for analysis. Additionally, we applied a bootstrap technique while accounting for the empirical probability distribution of the peptides within the TCR population.

For each sequence within a sample, the sequence length is determined, and sequences are grouped into lists based on their length. The total occurrences for each sequence length group within a sample are calculated, representing the total number of times sequences of a given length appear in that sample. Within each sample, we perform a ranking of group lengths based on their total occurrence counts—rank 1 is assigned to the most frequently occurring sequence length group. The ranks of each sequence length group are then summed across all samples. To determine the optimal sequence length for analysis, the sequence length group with the lowest rank sum across all samples is selected.

### Data preprocessing for HELP-TCR

After selection of length and bootstrapping, we are ready to transform the data into suitable format for our analyses. We performed 2 types of sample data transformation. As described in the Supplementary Materials, where we describe the formal models utilized in this study, both representations of data were based on frequencies of position-specific probability distributions over the alphabet of single amino acids or motifs of length 2 (pairs).

For the comparison of TCR repertoires using Wasserstein distance, each sample was transformed into a 2D array, representing positional information and the probability of each amino acid motif occurring at a given position. This transformation was applied to amino acid motifs of length 1 and 2.

For the deep learning module, each sample was transformed to consider pairs of amino acids in a position-wise manner, allowing for arbitrary gaps between the first and the second amino acid in the pair. The resulting data were structured into a multidimensional array, referred to as a tensor. After converting the data to the tensor format, we applied clustering of the motifs (pairs) based on similarity of abundance profiles and dimensionality reduction techniques to reduce the number of the motifs using a data-driven approach. Below, we describe our approach to data transformation prior to applying the deep learning model.

### Tensor data transformation

In our approach, we utilize data structures called tensors to represent the TCR population. This is somewhat similar to the analysis of images by means of deep learning, where the image is represented as a tensor with 3 “channels” (RGB) and in each channel is an *n*-by-*m* matrix of pixel intensities, where *n* and *m* are the vertical and horizontal number of pixels.

To recap, a tensor is a multidimensional array defined by dimensions of N×H×W, where H is the number of rows of the matrix, W is the number of columns, and N is the number of matrices of dimension H×W. We shortly describe the method of transforming peptide samples into tensors.

To illustrate this, set a pair of amino acids and consider a matrix $M={\left[M_{\mathrm{ij}}\right]}_{i,j=1,\ldots ,L},$, where L is the length of the peptide. Each element $M_{\mathrm{ij}}$ corresponds to the occurrence of a pair of amino acids (2 g) at positions i and j within the peptide, meaning that for a given pair of amino acids $M_{\mathrm{ij}}$ is the number of occurrences of the first amino acid from the pair occurring on position i and the second amino acid of the pair occurring on position j. The tensor structure is specifically designed to encode positional information for every possible motif of length 2, allowing for arbitrary gaps. In what follows, we merge these tensors across all peptides in the TCR repertoire to build a representation of the population of receptors—in other words, we create a tensor with dimensions P×N×L×L, where P is the number of peptides in the sample, $N\left(=400\right)$ is the number of amino acid pairs, L is the number of rows, and L is the number of columns.

In other words, for each peptide in the sample, a tensor representing the occurrence of amino acids is created.

The next step involves summing the matrices along the first dimension (P, number of peptides in the sample) and dividing the result by the number of receptors sequenced. This operation produces a tensor, where for each pair of amino acids, the element $m_{\mathrm{ij}}$ represents the probability of 2 amino acids occurring at positions *i* and *j*, respectively, within the peptide.

## Results

**Fig. 1.**
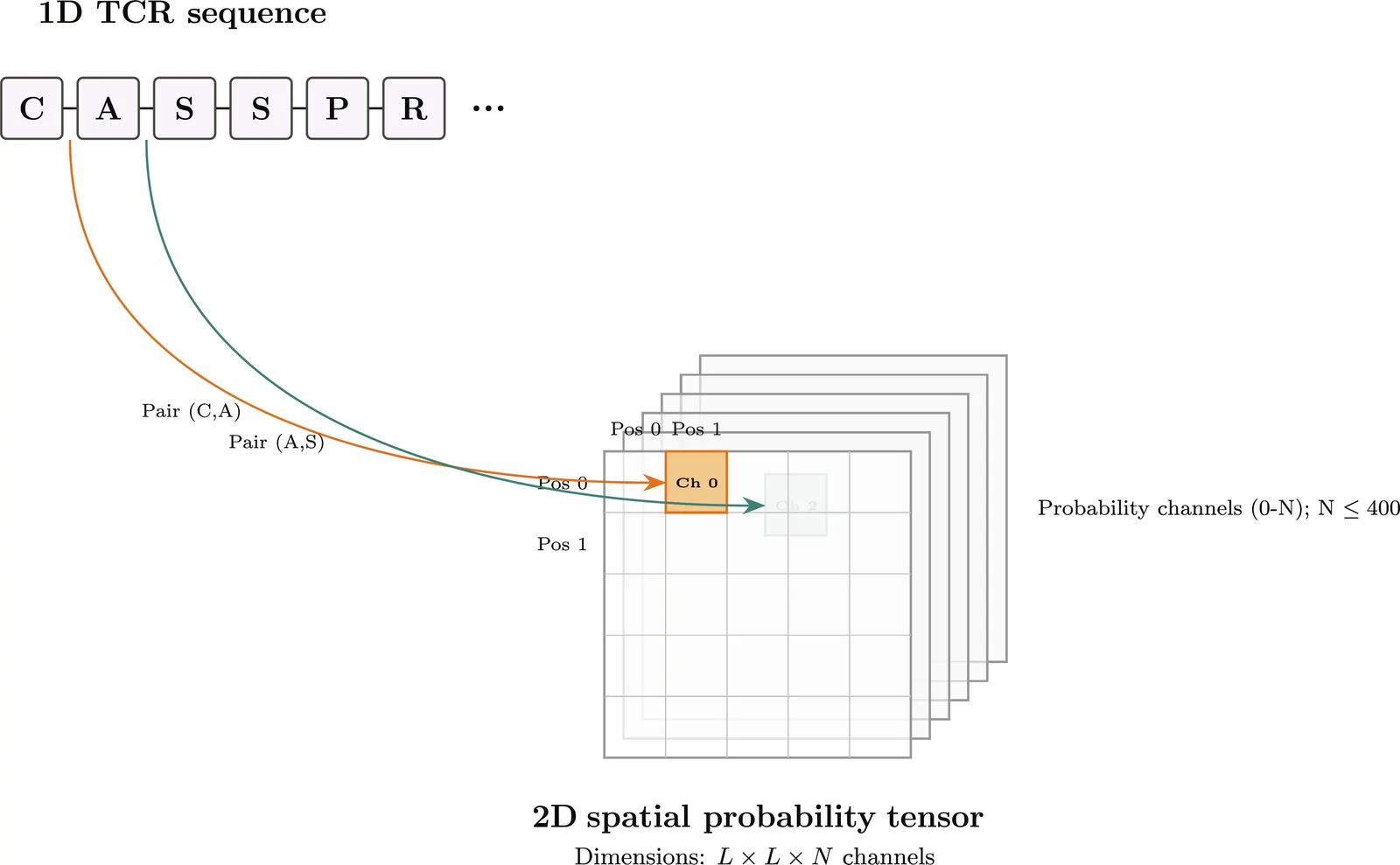
TCR representation workflow. Raw 1D TCRβ sequences are filtered by length and partitioned using a strict patient-level bootstrap protocol. Subsequently, pairwise spatial relationships are mapped and compressed via Louvain community detection into a multi-channel 2D probability tensor (L × L × N channels).

**Fig. 2.**
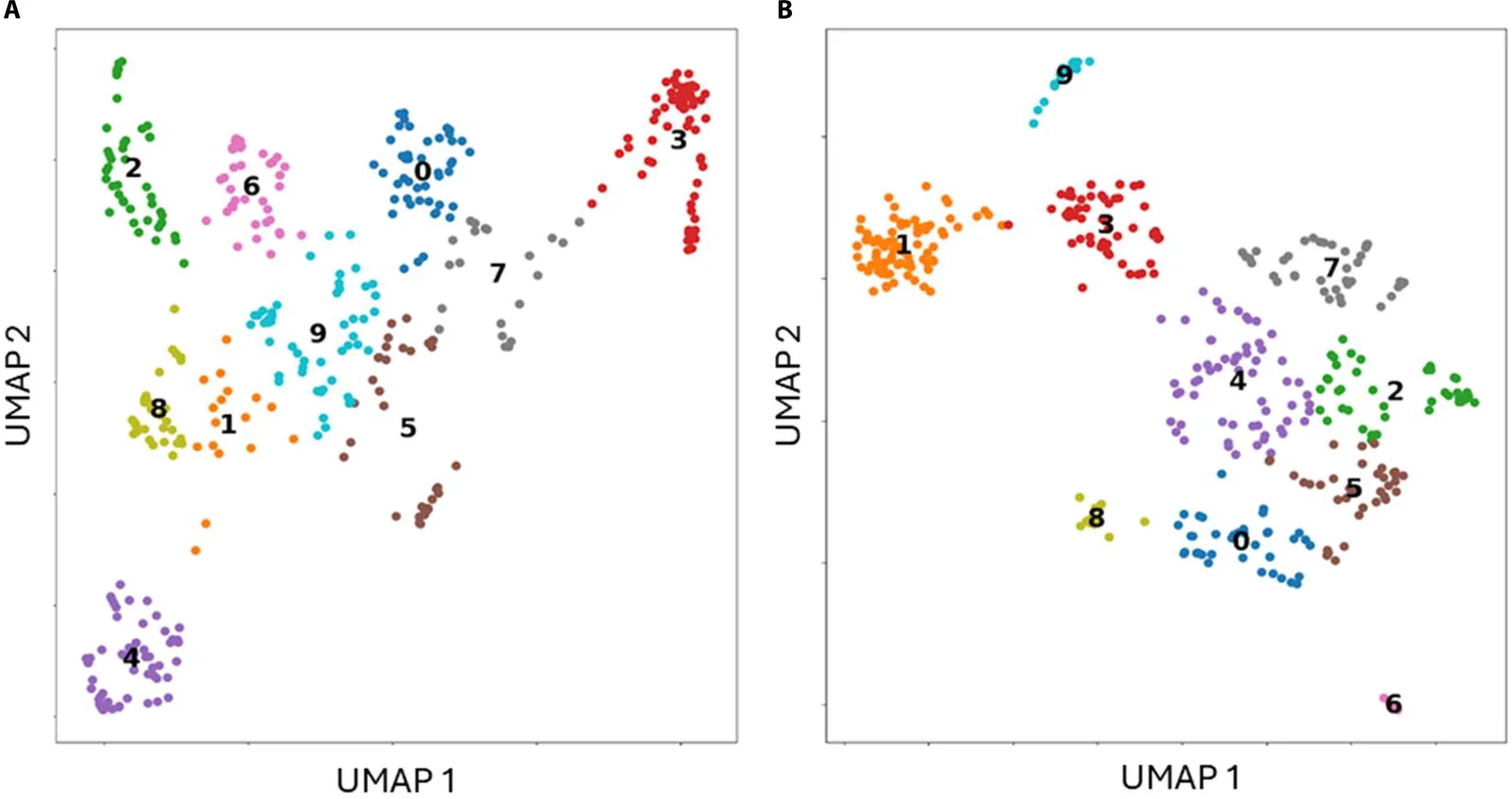
UMAP visualization of the distance matrix between amino acid pairs for an example sample transformed to a multidimensional array. Each data point represents matrix M corresponding to an amino acid pair occurring on a given position in a TCR sequence sample transformed to tensor. Colors indicate clusters obtained via clustering algorithm (KMeans) on UMAP-transformed data. (A) Clustered UMAP representation for an example sample belonging to class N (sample BMC-63-N_bts1). (B) Clustered UMAP representation for an example sample belonging to class T (sample MDA-1150-T_bts1_T).

In this section we present the results of the data preparation for HELP-TCR. Subsequently, we aim to compare our approach to the classification algorithm (DeepTCR) and a classifier based on embeddings generated by TCR-BERT. We also show how our methods may be applied to identify features most informative of the clustering of redundant motifs as well as supervised classification (Figs. 1 and 2).

### Dimensionality challenge - reducing the number of pairs

One of the challenges with this approach is the high dimensionality of data generated for each sample when transformed into tensors. To address this, the following dimensionality reduction procedure was applied.

For each sample, we first compute the distance between each matrix within the tensor and in this way from a distance matrix between the pairs of amino acids. The Frobenius norm of the difference was chosen as the measure of distance between matrices of the same size. For matrices $B=\left[B_{\mathrm{ij}}\right]$ and $A=\left[A_{\mathrm{ij}}\right]$, the norm ia mathematically expressed as follows:

$${\left|\left|B-A\right|\right|}_{F}=\sqrt{\sum_{i}\sum_{j}{\left(B_{\mathrm{ij}}-A_{\mathrm{ij}}\right)}^{2}}$$ (1)

Subsequently, we plug this distance matrix to the UMAP embedding algorithm—we use UMAP for the precomputed distances between points. In what follows, the KMeans algorithm on the result of the embedding is performed and configured with the number of clusters set to 10 and random state of 42. This setup was used in order to group the UMAP data points and assign labels to each group. The resulting labels are numerical values ranging from 1 to the number of clusters (in this case 10).

The next step in data processing involves creating an incidence matrix for each peptide sample. In order to do that, we use UMAP data points along with assigned labels. We then compare whether matrices for each tensor dimension co-occur in the same group. The resulting incidence matrix is denoted as $l=\left[l_{\mathrm{ij}}\right]$, where $l_{\mathrm{ij}}\in \left\{0,\;1\right\}$ and 0 means that 2 amino acid matrices do not belong to the same group and 1 means otherwise.

**Fig. 3.**
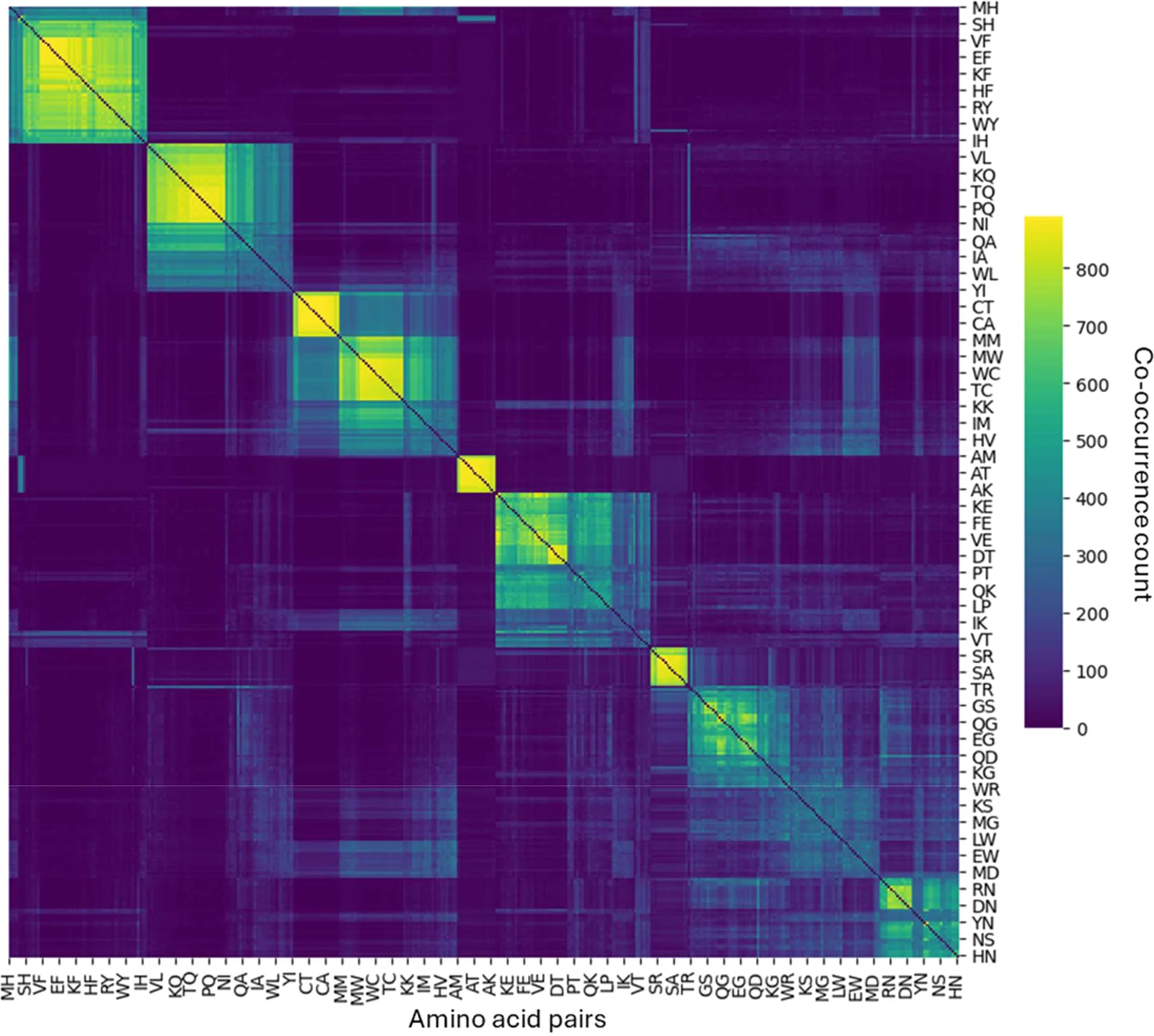
Cluster map based on the matrix of the summed of individual incidence matrices (generated via *k*-means clustering) across all samples. Each incidence matrix shows how many times the amino acid pair belonged to the same cluster provided by *k*-means clustering algorithm for every sample. The incidence matrices for each sample were computed and then summed up to represent amino acid pairs that appear in the same cluster for all the samples. The axes display amino acid pairs, limited to fewer than 400 for better visualization.

After computing incidence matrices for each peptide sample structure, we sum their values across all of the samples, shown on Fig. 3. The resulting matrix is denoted as D and may be expressed as:

$$D=\sum_{k}^{N}I_{k}$$ (2)

where N is the number of samples in the dataset.

The matrix D (which is an incidence matrix) is then used as input to the Louvain algorithm, which is designed for community detection in networks. The values from matrix D are treated as weights for a graph. This graph is clustered using the Louvain algorithm to identify patterns and groups. As the output of the Louvain algorithm, we obtained unique labels assigned across all tensor dimensions. In our case, the algorithm identified 5 groups and assigned labels accordingly. Additionally, the count of matrices belonging to each specific group for pairs of amino acids is as follows:

Labels: [0, 1, 2, 3, 4, 5],

Counts: [114, 88, 62, 62, 58, 16].

### Explainability of the Louvain cluster analysis

**Fig. 4.**
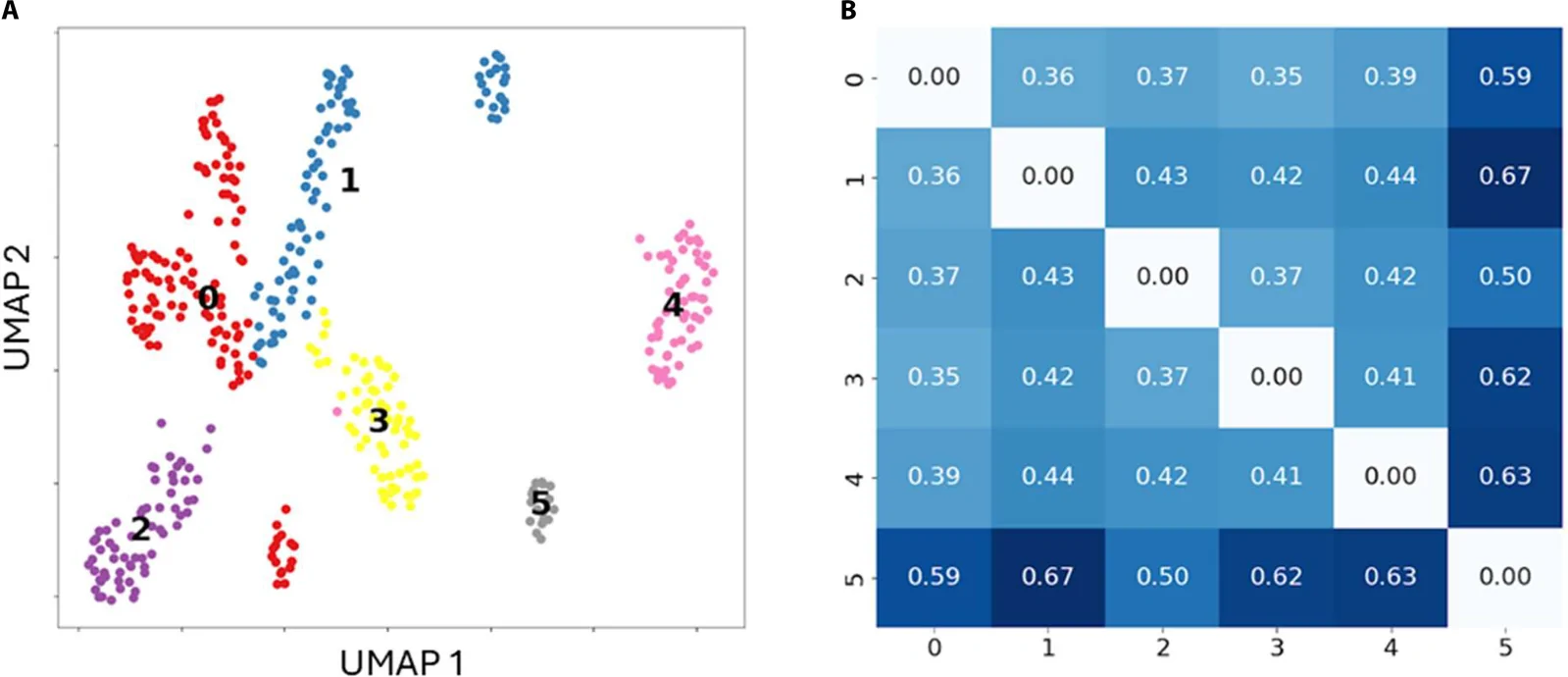
(A) An UMAP scatterplot of the summed incidence matrices, representing the co-occurrence frequencies of amino acid pairs within each cluster. Each data point corresponds to a specific amino acid pair, with the value indicating how often the pair appeared together across the sequences in the sample in a specific *k*-means cluster. Colors indicate clusters identified using the Louvain algorithm. (B) Heatmap representing distance matrix for Louvain clusters. As measure of distance, Earth’s mover distance was chosen. The Wasserstein distance shows how much clusters differ from each other.

We note that each cluster generated by the Louvain algorithm represents a group of amino acid pairs that have a similar pattern of occurrence in the TCRs. We analyzed the entropy of the distribution of the first and the second letter in the pair within each cluster. In this way, we assess the robustness of the data within each cluster and its ability to distinguish between the first and second amino acids in each pair (Fig. 4).

**Fig. 5.**
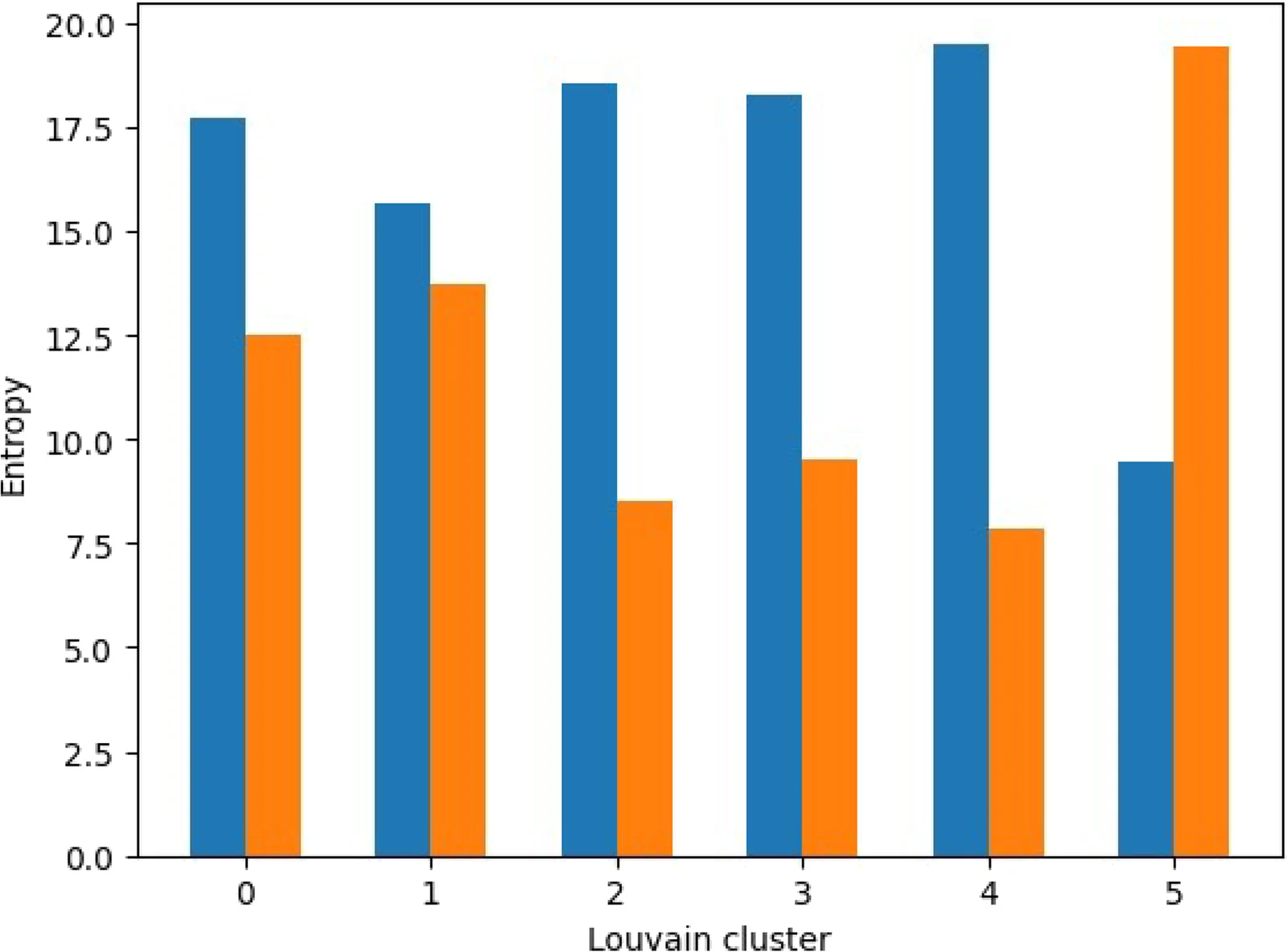
A bar plot representing the entropy of each Louvain cluster, calculated using amino acid pair counts as values. Each bar corresponds to the entropy value of a single position on the pair within a cluster. Colors of bars correspond to the order of amino acids in the pair. Blue represents the entropy of the distribution of the first amino acid in the pair, and orange represents the entropy of the distribution of the second amino acid in the pair.

From Fig. 5, we observe that cluster 5 exhibits the highest entropy for amino acids in the second position and the lowest entropy for the first position. This can be explained by the fact that cluster 5 is the smallest, containing only 16 amino acid pairs. Clusters 2, 3, and 4 show relatively low entropy for the second position but higher entropy for the first position, with the entropy being nearly equal across these 3 clusters. For the largest cluster, cluster 0, the entropy for the first position is similar to that observed in clusters 2, 3, and 4, but the entropy for the second position is higher. In cluster 1, the second-largest cluster, we see that the entropy for the first position is greater than that for the second position, although the difference is not substantial.

In what follows, we calculate the multinomial distribution of counts of amino acids on the first and the second position of the pair for each Louvain cluster. From this “null” distribution, we perform a parametric bootstrap procedure to evaluate the variability when comparing these distributions between clusters. In this way, we ask the question on the specificity of amino acids for a given cluster. In what follows, a Random Forest classifier was applied to the data, separately for the first and second positions—with the label of the cluster being the “dependent variable”. The main objective was to interpret the per-sample classification results of the Random Forest model to identify which amino acids had the greatest impact on class membership. To achieve this, we computed the Gini index and SHapley Additive exPlanations (SHAP) values [15,16].

We aim to further explain the clustering of pairs of amino acids and propose a visualization of the distribution of each “important amino acid” within all clusters. For the purpose of the present paper, we have chosen amino acids that are common for both feature importance based on Gini index and SHAP values.

As mentioned earlier, the entropy for clusters 2, 3, and 4 is quite high for the first position in the amino acid pair. This explains why the violin plots for those amino acids have such low variability, reflecting the low number of occurrences in those clusters. The high importance of these amino acids in classification can be easily attributed to their high frequency of occurrence in clusters 0, 1, and 5.

**Fig. 6.**
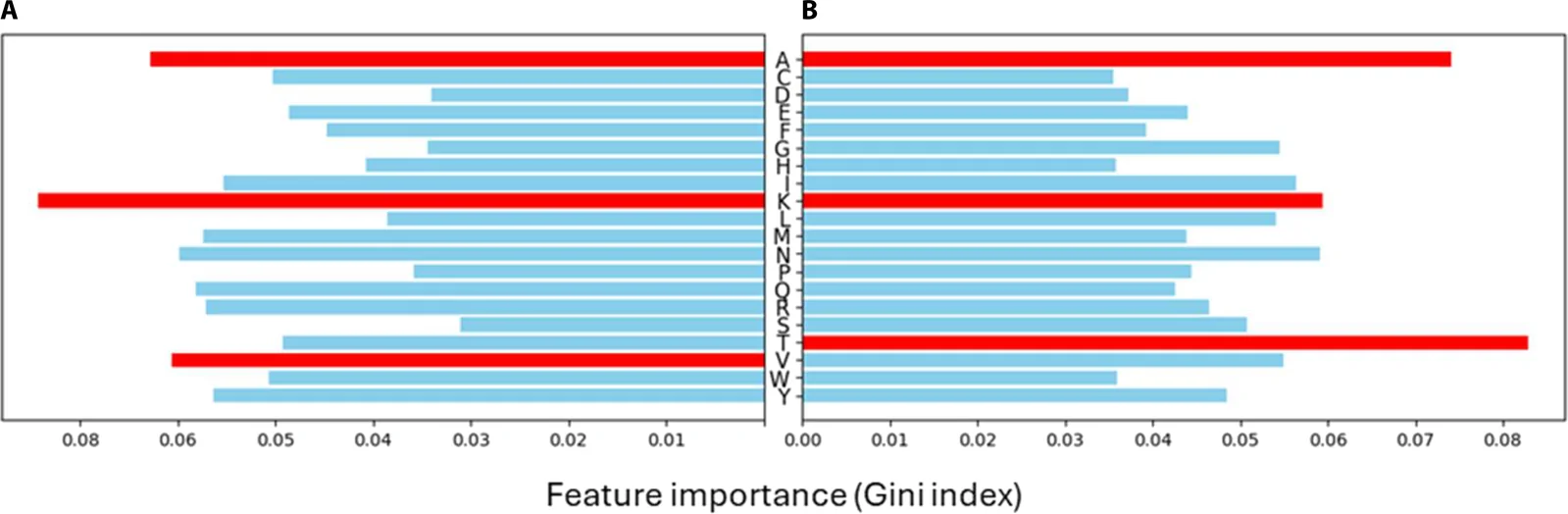
A bar plot representing amino acids that were important for the classifier applied between Louvain clusters. The chosen algorithm is Random Forest classifier, with the Gini index used as the measure of importance. The top 3 most important amino acids are highlighted in red. The *X* axes show the value of importance represented as Gini index, and the *Y* axes show the amino acids used as features for supervised classification. (A) Feature importance for the first letter in amino acid pairs in Louvain clusters. (B) Feature importance for the second letter in amino acid pairs in Louvain clusters

**Fig. 7.**
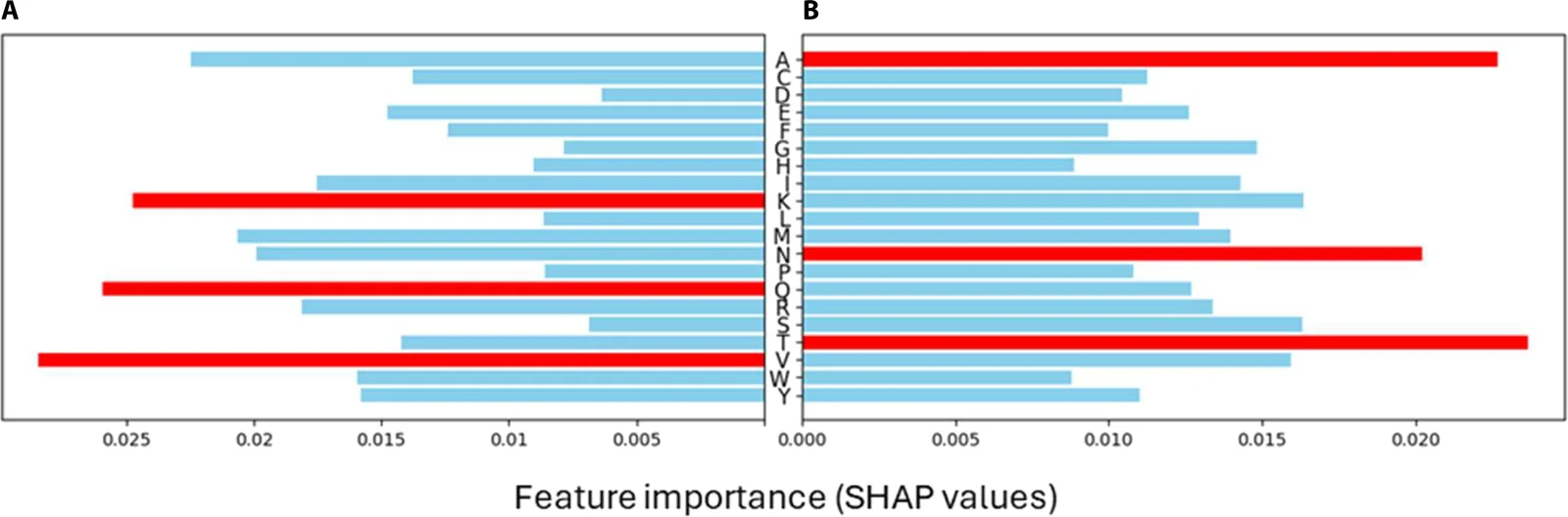
A bar plot representing the importance of amino acids in supervised classification with applied Random Forest classifier. The impact of each amino acid on the classification is shown with computed SHAP values. The top 3 most important amino acids are highlighted in red. The *X* axes show the SHAP values, and the *Y* axes show the amino acids used as features to perform supervised classification. (A) Feature importance for the first letter in amino acid pairs in Louvain clusters. (B) Feature importance for the second letter in amino acid pairs in clusters provided by Louvain clustering algorithm.

**Fig. 8.**
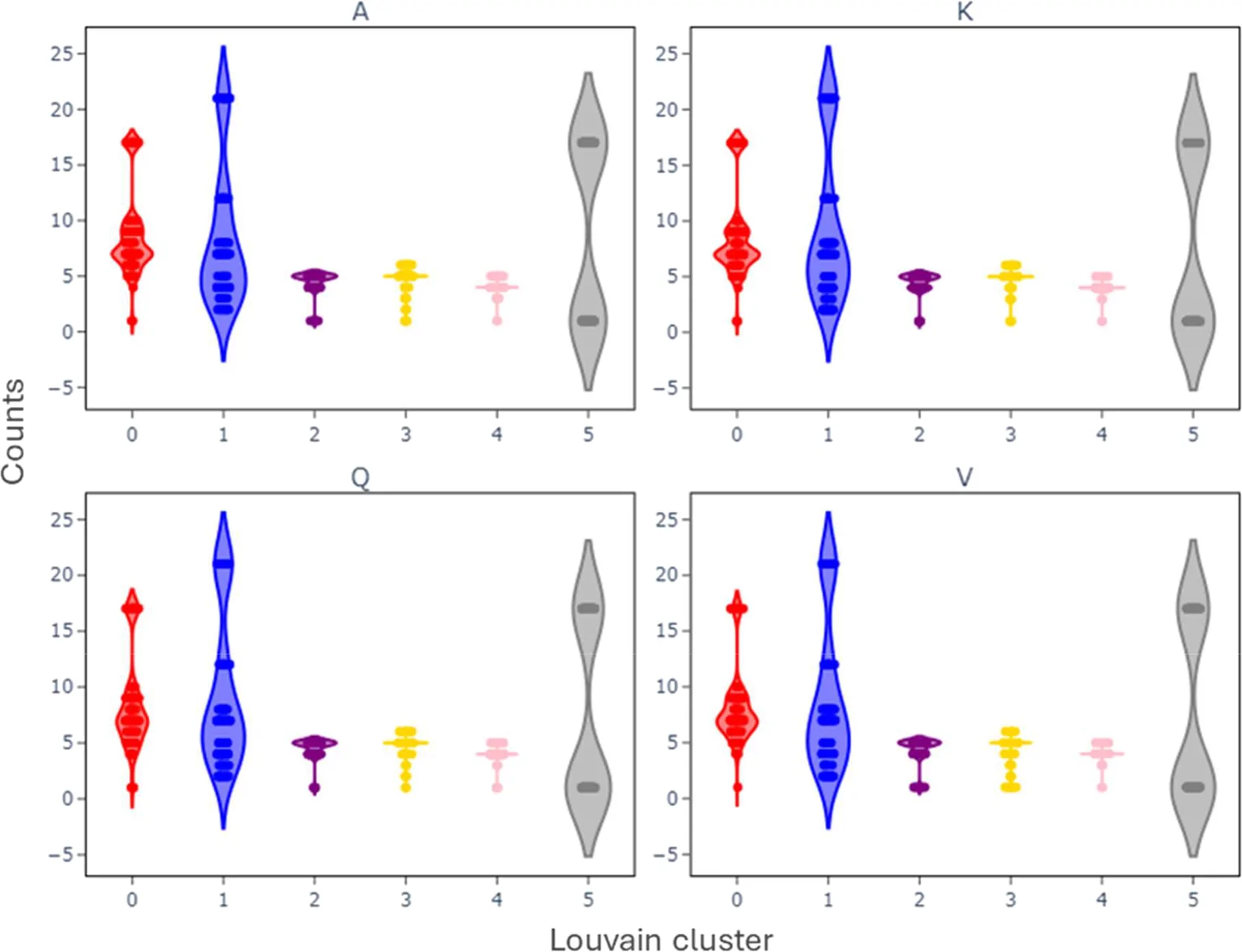
A violin plot for each amino acid with a high importance value corresponding to the first position in a pair. Each violin plot represents a different cluster, illustrating the distribution of amino acid counts in each cluster. *X*-axis labels represent the label of the cluster provided by the Louvain clustering algorithm.

**Fig. 9.**
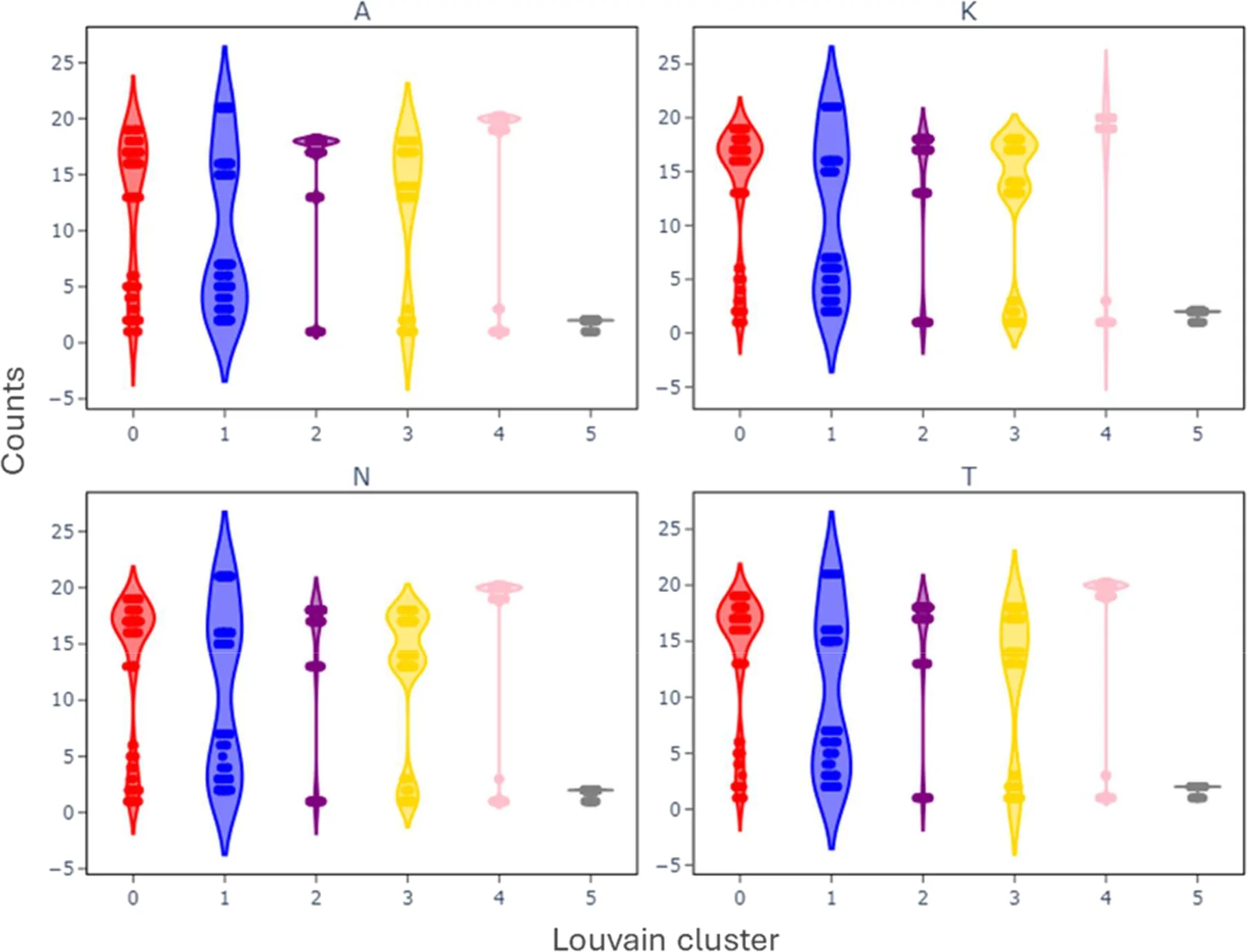
A violin plot of 4 most informative (with respect to the amino acids with a high importance value corresponding to the second position in a pair). Each violin plot represents a different cluster, illustrating the distribution of amino acid counts at the second position. *X*-axis labels represent the label of the cluster provided by the Louvain clustering algorithm.

For the second position, the entropy in cluster 5 is high, which is evident in the violin plots. The amino acids that are important for other clusters are almost absent in cluster 5, further highlighting the distinctiveness of this cluster (Figs. 6 to 9).

### Comparison of TCR repertoires by means of Wasserstein distance

Due to the economy of space, we do not present the results of the Wasserstein distance analysis for single amino acids and amino acid pairs with no position-wise dependence. The reader may test these functionalities, for the example dataset provided in the library. Instead, we focus on the results of the analysis of the abundance of amino acids as well as pairs of amino acids represented in the position-wise dependent manner.

**Fig. 10.**
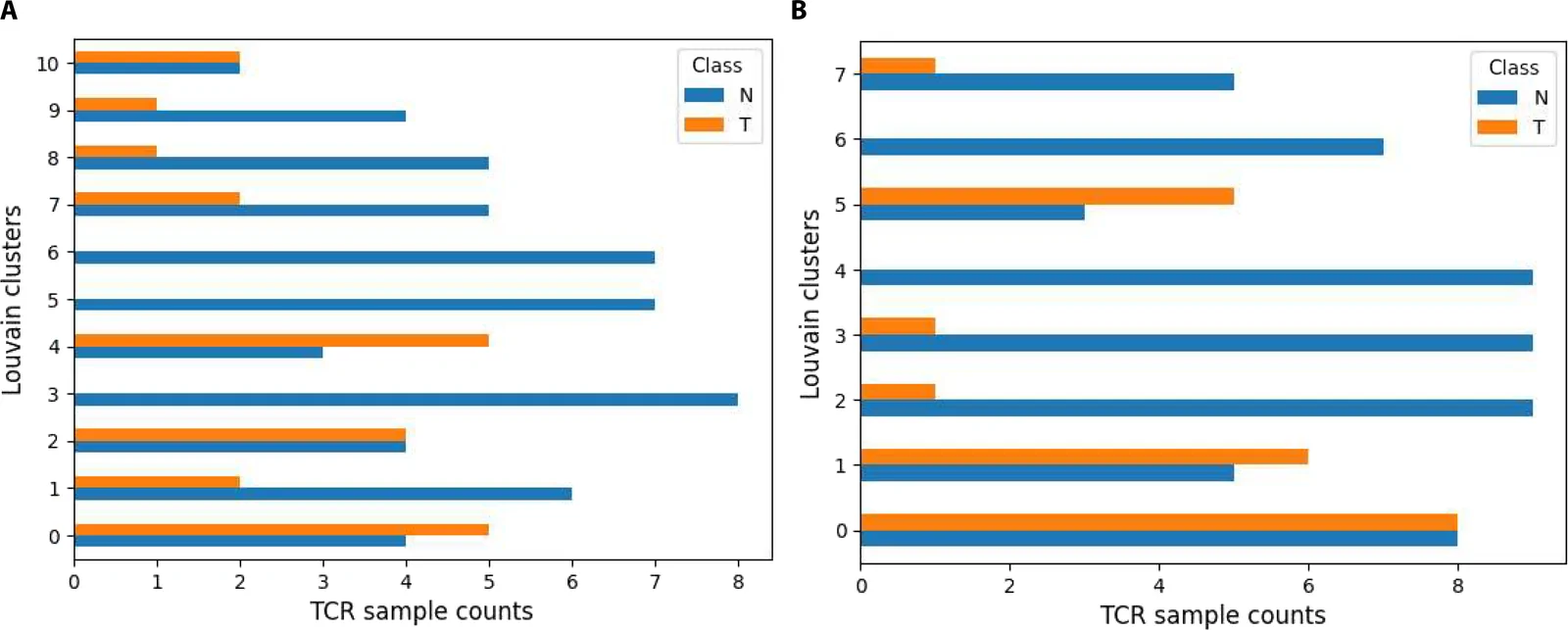
The bar plot represents the number of samples belonging to classes “N” (normal) and “T” (tumor) within each Louvain cluster for the analysis based on the Wasserstein distance. (A) Performance on a single amino acid position-wise basis. (B) Performance on the position-wise distribution of amino acid pairs.

Firstly, we performed unsupervised learning to cluster the TCR populations using the Wasserstein distance matrix based on the position-wise distribution of single amino acids. We start with calculating the pairwise distances and represent the matrix as a graph. In what follows, we prune the edges of the graph using ARACNE [17] method and cluster the pruned graph by means of the Louvain method [18]. As a result, we obtain 11 clusters, which are presented in Fig. 10. We note that the prevalence of T versus N samples in these clusters allows for mild discrimination between the T and N samples, as clusters 3, 5, and 6 contain exclusively samples tagged as N and clusters 0 and 4 contain more T samples compared to N.

We applied the same approach to cluster the TCR populations using the Wasserstein distance matrix based on the position-wise distribution of amino acid pairs. As a result, we obtained 8 clusters, which are represented in Fig. 10. Notably, these clusters allow for mild discrimination between the T and N samples, as clusters 4 and 6 contain only N samples, while clusters 2, 3, and 7 show a clear predominance of N samples over T samples.

### Side-by-side comparison between HELP-TCR, TCR-BERT, and DeepTCR

In our study, we compared the proposed approach with other existing methods for TCR repertoire classification (that is, per-sample classification of TCR ensemble) based on language processing and deep learning. In this short section, we present the main results of this comparison. We defer the detailed descriptions of the results for each software to subsequent sections of our manuscript.

**Fig. 11.**
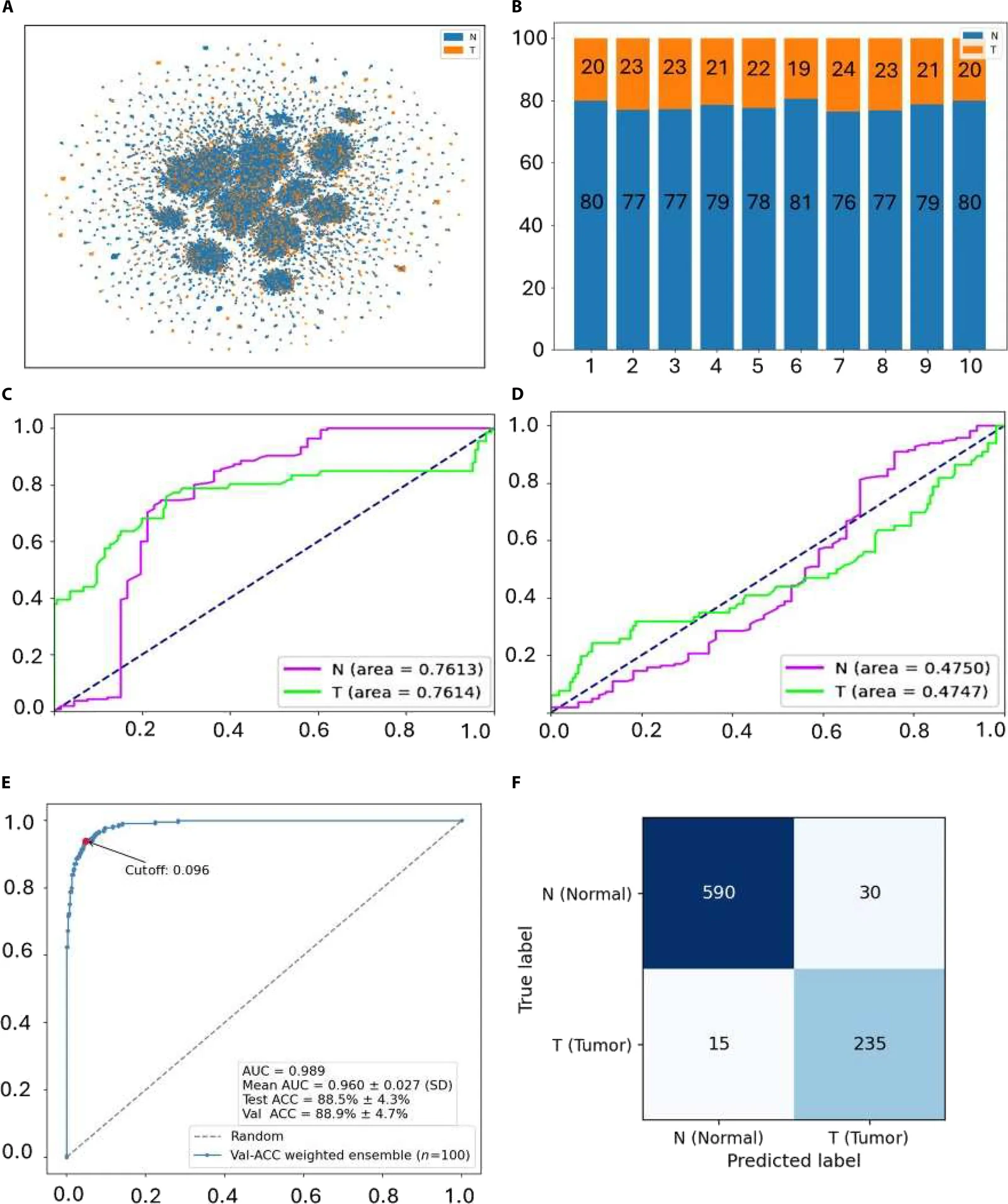
Comparison of the results for HELP-TCR, TCR-BERT, and DeepTCR. (A) TCR data representation using embeddings generated by the TCR-BERT software. (B) Proportion of samples classified as tumor or normal within the largest clusters identified by TCR-BERT. (C) ROC curve illustrating the performance of DeepTCR, with an AUC of 0.76. (D) ROC curve illustrating the performance of DeepTCR, with an AUC of 0.47. (E) ROC curve of HELP-TCR, demonstrating an AUC of 0.96. (F) Confusion matrix for HELP-TCR, built using TCR-transformed data.

We analyzed the same dataset that consists of 1,000 bootstrap samples from the TCR repertoires by means of unsupervised classification with the aid of TCR-BERT and found 10 main clusters for which we conducted a post hoc comparison of the fraction of T versus N samples. The results of the clustering as well as the bar plot describing the fraction of T versus N samples in each cluster are presented in Fig. 11A and B.

We also applied DeepTCR to the same samples to get supervised classification of TCR repertoires. The results of classification as well as receiver operating characteristic (ROC) curve and AUC describing how well the DeepTCR model performs on the dataset are presented in Fig. 11C and D. In comparison, we present our results of applying modified ResNet-18 neural networks to processed TCR samples with respect to short motifs-pairs of amino acids.

The results of per-sample classification using our algorithms and approaches are presented with a ROC curve with AUC and a confusion matrix displayed on Fig. 11E and F.

**Fig. 12.**
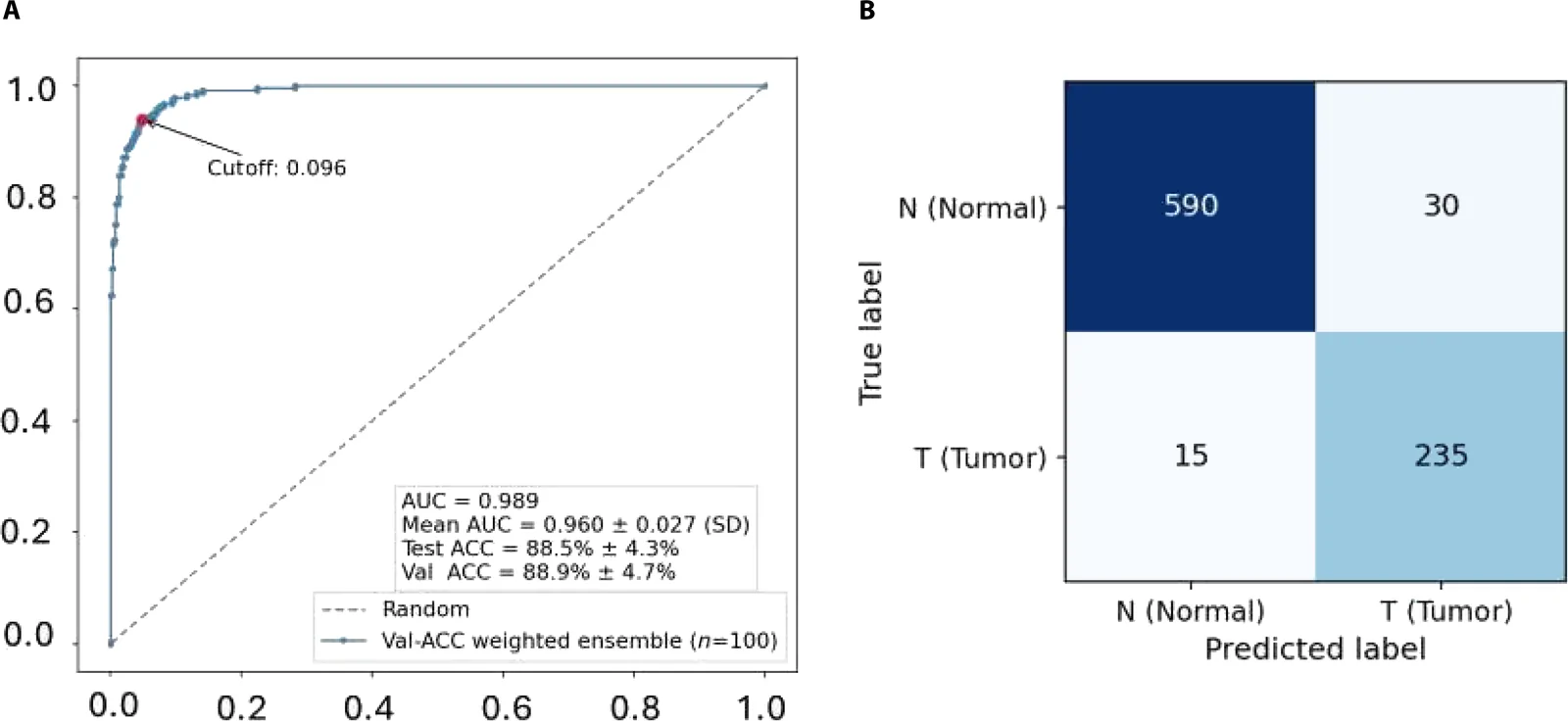
(A) ROC curve for HELP-TCR. The AUC is shown on the legend. (B) Confusion matrix of HELP-TCR performance (with an accuracy score of 88.76%).

Additionally, we note that the comparison between these 3 methods involves fundamentally different analytical paradigms. HELP-TCR and DeepTCR operate as supervised classifiers with direct access to sample labels during training, whereas our TCR-BERT analysis relied on unsupervised embedding followed by UMAP visualization and Leiden clustering. This choice reflects the typical usage mode of TCR-BERT as a representation learning tool rather than an end-to-end supervised classifier. Consequently, the TCR-BERT results should not be interpreted as evidence that the model intrinsically fails at discrimination, but rather that its pretrained embeddings, without task-specific fine-tuning, do not separate the classes in the latent space for this particular dataset. A supervised fine-tuning of TCR-BERT for repertoire-level classification remains an interesting direction for future work. To further probe the discriminative capacity of TCR-BERT without fine-tuning, we implemented a simple supervised baseline in which per-sequence embeddings from the last hidden layer were mean-pooled across all TCRs belonging to the same sample, producing a single repertoire-level representation. A logistic regression classifier trained on these vectors under stratified 5-fold cross-validation achieved an AUC of 0.58, confirming that the pretrained embeddings do not carry sufficient task-specific signal for tumor versus normal discrimination at the repertoire level in the absence of fine-tuning (Fig. 12).

### Explainability of the HELP-TCR results

In HELP-TCR, we performed deep learning-based per-sample classification on the data preprocessed as described in the Data preprocessing for HELP-TCR section. We have also modified the architecture of the ResNet deep learning model to increase the number of allowable input channels (as we do not impose the rule that we need 3 clusters of amino acid pairs). After applying clustering and dimensionality reduction techniques on the TCR repertoires, we reduced the number of output dimensions of the tensor. The remaining matrices within the tensor correspond to the number of channels that our deep learning model is designed to process.

As the base architecture, we utilized ResNet-18, modifying the number of neurons per layer to 8, 16, 32, and 64 instead of the conventional 64, 128, 256, and 512. This adjustment was motivated by the nature of TCR sequences, where a lower number of neurons was sufficient to capture the key distinguishing features of the data while also facilitating a more efficient training process.

To enhance model robustness, we applied a bagging technique to the model weights. Specifically, we trained 100 neural networks, each initialized with a different random seed for data splitting. The trained models were then evaluated on the validation set, and the predictions from all networks were aggregated for further analysis.

For each network, we computed the model output for every sample in the validation set. We then determined the predicted class by identifying the index of the maximum value in the model output. The weights were then computed as follows:

$$a_{0}=\sum_{k=1}^{100}a_{k};w_{k}=\frac{a_{k}}{a_{0}}$$ (3)

where $a_{k}$ is the accuracy of the kth ResNet18 model on the validation set and $w_{k}$ is the weight for the kth ResNet18 model.

Note that the weights 𝑤*_k_* are determined solely from validation set performance and are fixed prior to any evaluation on the test set. The ROC curve was plotted based on the weighted sum of the predictions from each ResNet-18 on the test set. In other words, we take the weights $w_{k}$ for the kth network and define a weighted average of the logit transformed posterior probabilities of classification as:

$\text{logit}\left(p^{i}\right)=\sum_{k=1}^{100}\text{logit}\left(p_{k}^{i}\right)\cdot w_{k}^{i}$, for ith sample, i=1,…,N samples in the test dataset. Using the optimal cutoff value obtained from the AUC computation, we plotted the confusion matrix.

To assess the robustness of our classifier, we trained 100 ResNet-18 models independently, each with a different random seed governing the train/validation/test split. Across these 100 runs, the mean AUC was 0.960 ± 0.027 (SD), with an empirical 95% confidence interval of 0.889 to 0.994, confirming stable and consistently high discriminative performance. Mean test accuracy was 88.5% ± 4.3%, and mean validation accuracy was 88.9% ± 4.7%, indicating that performance estimates generalize consistently across splits with no systematic gap between validation and test evaluation. To further exploit the ensemble of 100 trained models, we constructed a validation-accuracy weighted ensemble, combining each model’s predicted probabilities proportionally to its validation accuracy (weights summing to 1.0, ranging from 0.008 to 0.011, reflecting the narrow spread of model quality across runs). The resulting ensemble achieved an AUC of 0.989, demonstrating that aggregating predictions across diverse random splits substantially reduces variance and yields a more reliable decision boundary than any single model. The optimal classification threshold for the ensemble was determined by maximizing Youden’s *J* statistic on the validation set, yielding a cutoff of 0.096 [true positive rate (TPR) = 0.940, false positive rate (FPR) = 0.048].

**Fig. 13.**
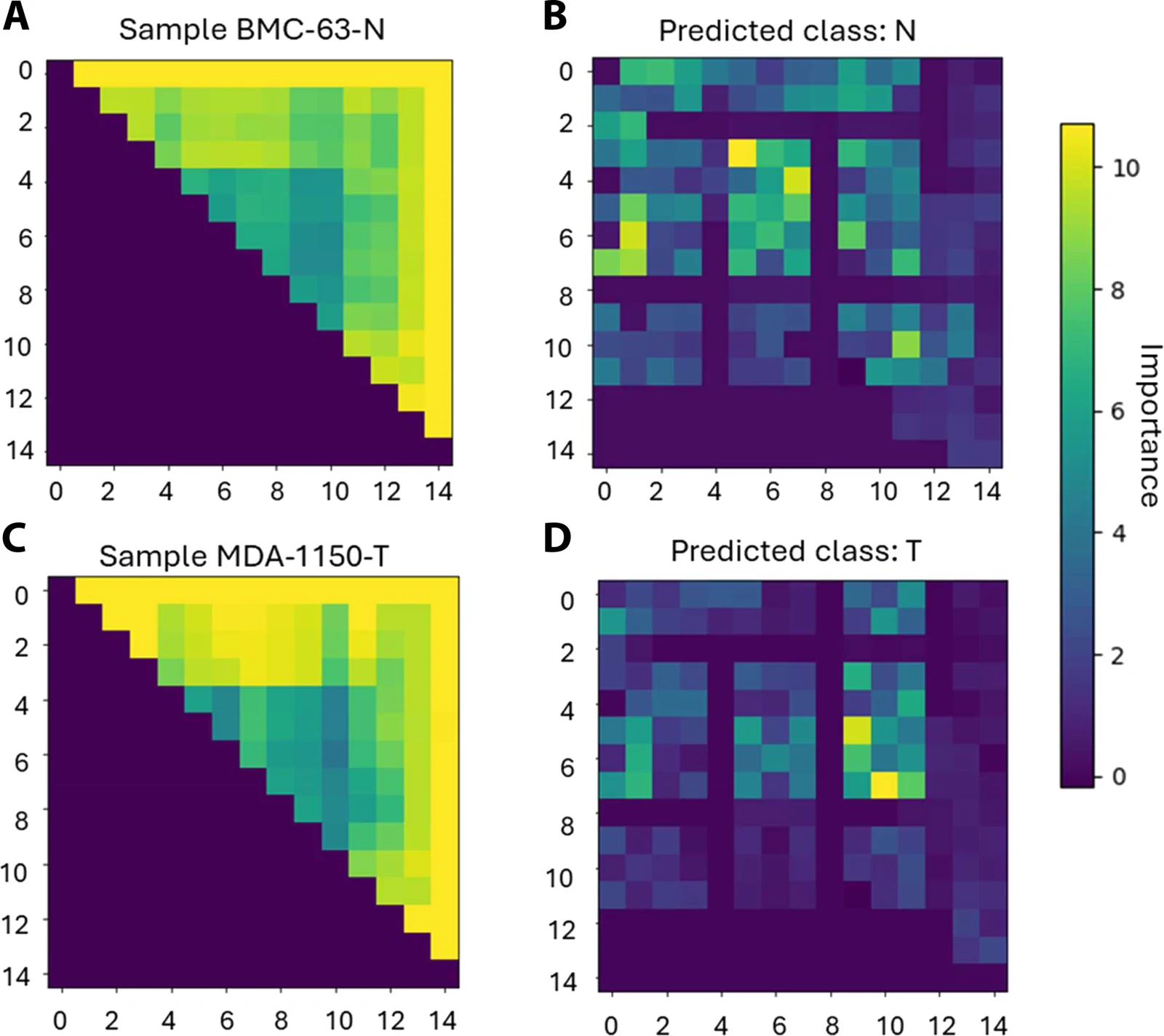
Saliency maps illustrating the performance of our algorithm per sample. (A) Transformed sample BMC-63-N, belonging to class N (normal), and its corresponding saliency map on (B). In the saliency map, color intensity indicates the importance of each region in the model’s prediction for classification into class N (normal) and class T (tumor). (C) Transformed sample MDA-1150-T, belonging to class T (tumor), and its corresponding saliency map (D).

We further proposed an approach based on saliency maps to make our approach based on ResNet-18 explainable. For each sample, we provide a saliency map, which highlights the features that were most important for the algorithm’s decision-making process. We provide an example of such maps in Fig. 13. The top panel presents the saliency map for the class N sample. In this case, we see that the amino acids present in the middle part of the TCR chain were most informative for prediction. For the second sample shown in the bottom panel, we see that the saliency map indicates that the pairs of amino acids present at position 9–11 × 5–7 are most informative for classification of this sample to the T group.

**Fig. 14.**
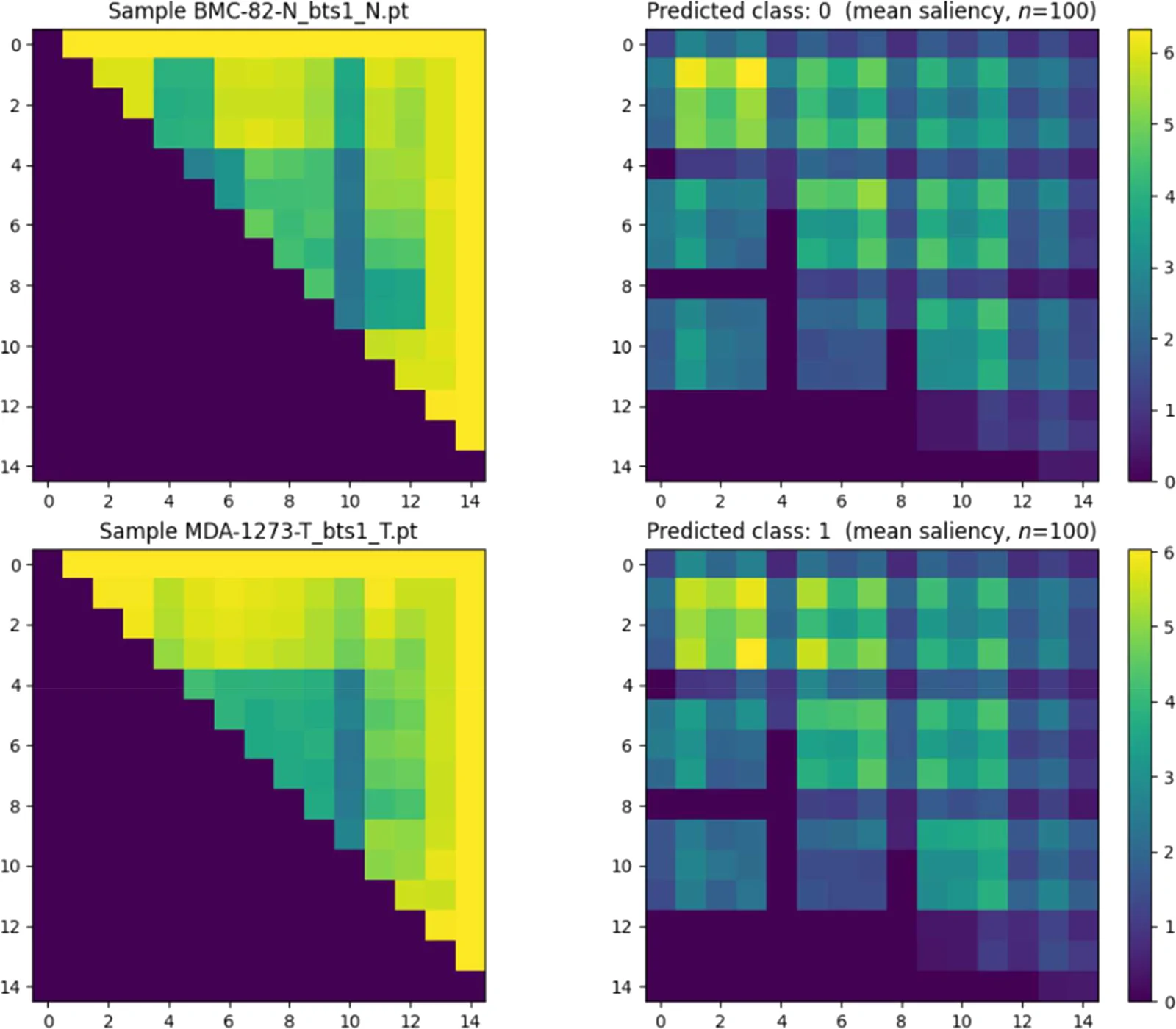
Averaged saliency maps computed across 100 independently trained ResNet-18 models for 2 held-out samples fully excluded from training, validation, and test sets. The left column shows the input tensor representations; the right column shows mean gradient-based saliency maps indicating which positions most influenced the model’s prediction.

We also provide the corrected saliency maps, mean across all 100 models. The figure shows averaged saliency maps computed across 100 independently trained ResNet-18 models for 2 held-out samples that were fully excluded from training, validation, and test sets. We provided the result as Fig. 14. The left column shows the input tensor representations, and the right column shows the mean gradient-based saliency maps indicating which positions most influenced the model’s prediction.

The positional regions highlighted by saliency analysis, Fig. 13 (positions 5–7 and 9–11 in the 15-mer CDR3 sequences), correspond to the central loop of the CDR3 region, which is the primary determinant of antigen specificity in TCRβ chains. This region makes direct contact with the peptide presented by MHC molecules (as inferred by the stable state crystal structures), and its amino acid composition is known to influence antigen recognition. The observation that amino acid pairs spanning these positions are most informative for distinguishing tumor-derived from normal TCR repertoires is consistent with the hypothesis that tumor-reactive T cells exhibit characteristic CDR3 loop features. A detailed structural mapping of these motifs to known tumor-associated TCR features represents a promising direction for future investigation.

## Conclusions

We presented a method for comparison of TCR repertoires, that is, per-sample classification of TCR ensemble based on amino acid motifs. Our core contribution is a data-driven approach that transforms TCR sequences into multidimensional tensors, enabling the application of advanced per-sample classification algorithms or per-group detection of informative features. By performing dimensionality reduction on this tensor structure, we substantially reduce the likelihood of false-positive associations. Unlike existing software solutions, our method provides a fully explainable framework for TCR analysis while maintaining competitive—often superior—predictive performance. The defining differentiator between our approach and tools like DeepTCR or TCR-BERT is interpretability: We explicitly map the clustering process via amino acid alphabet transformations and dimensionality reduction. Furthermore, we provide sample-specific saliency maps offering insights into the neural network’s decision-making process. We gather the features of the algorithms used in the comparative study in Table 1.

**Table 1.** Table comparing the basic parameters and functionalities of HELP-TCR to available software for T cell receptor analysis

Tool name	HELP-TCR	DeepTCR	TCR-BERT
Criterion			
Explainability	Yes	No	No
Classification	Yes	Yes	No
Embedding	Yes	Yes	
GPU	Yes	Yes	
Multiple GPU	Yes	No	
Software	PyTorch	TensorFlow	HuggingFace
Training time	~0.33	~16 min	~15 min
AUC	0.969	0.762	No

The spatial probability tensor framework introduced in this study presents a highly adaptable approach to TCR representation. Yet, our empirical validation was deliberately constrained to TCRβ sequences of a single, fixed CDR3 length. This specific length was selected as the most frequently occurring across all samples, providing the most representative and statistically robust subset for initial model training. By strictly controlling the geometric complexity in this foundational phase, we ensured that the network’s behavior—and the resulting explainable artificial intelligence (XAI) saliency maps—remained highly tractable, allowing us to definitively validate the extraction of true structural motifs without the confounding variables of length discrepancies. Importantly, the underlying mathematical transformation of 1D sequences into 2D spatial interaction grids is theoretically agnostic to chain size or type. Yet, unlike standard 1D sequence alignments, transforming TCRs into 2D probability matrices means that varying lengths fundamentally alter the dimensions of the spatial interaction manifold. Similarly, incorporating paired αβ chains requires expanding the tensor to capture complex interchain cross-interaction matrices. Because scaling this spatial topology introduces significant computational complexity, it was imperative to first validate the foundational model, convergence, stability, and XAI saliency extraction on a curated, controlled, representative baseline. Having successfully demonstrated that this architecture can isolate informative motifs within a standardized grid, adapting the spatial mapping to handle dynamic interpolation for variable lengths and cross-chain αβ pairing remains the primary objective for our future work.

In a comparative analysis of 3 methods applied to the same TCR dataset, HELP-TCR achieved an AUC exceeding 96%. In contrast, the ROC curves generated by DeepTCR demonstrated significant variability depending on the data split ratio, with AUC values ranging from 0.475 to 0.761. Our analysis of TCR-BERT involved generating embedding representations followed by visualization. Notably, the clusters observed in the TCR-BERT embedding space were heterogeneous, containing a mixture of samples from both the classes. An investigation of the 10 largest clusters revealed minimal variation, with approximately 20% of sequences belonging to the T class and 80% to the N class. The high performance of HELP-TCR (AUC 0.969) is bolstered by our implementation of bagging, ensuring robust discrimination.

Our data transformation reflects positional information and accounts for the observed likelihood of amino acid pairs occurring within a sample. The embedding—dimensionality reduction—identifies clusters of these pairs, serving as the basis for reduction of the alphabet of common amino acids. To accommodate datasets with a channel depth exceeding standard RGB image analysis, we adapted the ResNet architecture, demonstrating its feasibility for high-dimensional immunological data. We demonstrated that our representation of the data is versatile—it supports not only deep learning applications but also standard machine learning techniques, such as classification based on the Wasserstein distance.

HELP-TCR is available on GitHub, where we plan to expand its capabilities through new modules and user feedback. To bridge the gap with classical TCR analysis, we also include a visualization module for generating logo plots. We also make the code for HELP-TCR available at https://zenodo.org/records/17944547.

While a full web server is planned for a future release, the current open-source Python package includes comprehensive documentation, example datasets, and reproducible workflows that enable users to apply HELP-TCR to their own data with minimal setup.

### Future work

The proposed transformation of CDR3 loop of the TCRs into 2D multi-channel spatial (sample level) probability tensors provide a framework that extends beyond binary comparisons of per-sample repertoires. The direction for future research lies in using this representation for structural biomarker discovery. As demonstrated by our gradient-based saliency mapping, in the controlled setting of similar training–testing splits, we can detect robust signals that are biologically meaningful, making our approach fully explainable. Our future work, which is already ongoing, focuses both on mimicking interesting biological or clinical scenarios (like mixing samples in bootstrap to simulate longitudinal TCR monitoring) and on building custom artificial intelligence (AI) engines that are suitable for deciphering meaningful signal from tensors with a much lower dimension than used traditionally in computer vision.

For demonstrational purposes, we have developed both the scenario with mixed samples in the training set and original samples in the testing set, as well as an alternative AI engine—a “Gentle ResNet”, both of these we describe below.

We first evaluate model robustness by deliberately introducing a “mixing split”. By mixing samples in the training phase, we demonstrated that the original almost trivial ResNet architecture can still successfully extract the underlying structural features. What is probably much more interesting is that this specific experimental design (mixing samples) mimics a highly relevant clinical scenario: the longitudinal embedding and tracking of highly similar, evolving clones from the same patient across multiple time points. This proves the feasibility of applying our approach to cases when only selected TCRs undergo clonal expansion and the repertoire itself remains stable (public receptors tied to ambiguous antigens). Here, we also introduce a pivot in the ensemble learning phase. To fully utilize the variance captured across the 100 independent bootstrap iterations, our aggregation strategy utilized a stacking meta-learner. We do this as an alternative to the soft voting approach initially used in the manuscript. We use logistic regression as the meta-classifier and train it over out-of-fold (OOF) predictions. In this way, we believe that we were able to average-out the stochastic noise inherent to specific random splits and produce a robust consensus prediction of AUC = 0.98.

For the second extension, we implemented a rigorous group shuffle split, where no replicate from the original samples overlaps between the training, validation, and testing sets. This guarantees that all augmented tensors derived from a single biological sample remain strictly isolated within either the training, validation, or testing fold. We wish to note that this strict isolation together with the original ResNet architecture may introduce severe data “clumping”—i.e., a case where the model is likely to overfit to a patient-specific clonal dominance rather than the underlying tumor motif. This is due to the fact that the use of the standard deep convolutional neural network (CNN) often leads to overfitting in low-diversity training set scenario. To bypass this in HELP-TCR, we add a custom-designed “G-ResNet” (Gentle ResNet) architecture, which is built as follows:

• Layer 1: initial convolutional kernel (7 × 7) to capture broad, spatial interaction motifs across the probability clusters, immediately stabilized by robust batch normalization and ReLU activation.
• Layers 2 and 3: shallow residual blocks (paired with aggressive dropout)—all of this prevents the network from memorizing patient-specific profiles
• The macro-skip connection: Because of the massive dropout, we implemented a global residual connection—so the network does not flatten the data too much. Here, the original input tensors (raw inputs) are skipped past all intermediate convolutional blocks and added directly back into the deep feature maps. This trick allows the network to use the raw biological signal in the output.
• Output: Global average pooling is performed into a final fully connected layer.
• Optimization: Optimization was performed using the dynamic weighted cross-entropy loss, recalculating inverse class weights for every independent bootstrap fold and ADAM as the engine.

We evaluate 100 patient-isolated bootstraps in this setting. Here, we utilized an OOF rank-harmonized ensemble—converting raw predictive probabilities to percentile rankings before consensus averaging, resulting in AUC = 0.92

In this extended setting, using gradient based, channel-specific saliency maps, we are still able to find robust signal affecting the discrimination. In other words, we can isolate the precise geometric coordinates and specific amino acid probability clusters (e.g., C-terminal anchor motifs) that drive tumor recognition. We aim to scale this explainability pipeline across diverse oncological and autoimmune datasets to systematically catalog structurally grounded reactive motifs that are otherwise invisible to sequence alignment. The robustness of this tensor representation makes it suitable for longitudinal clinical monitoring. Future applications will deploy this methodology to track the spatial evolution of patient TCR repertoires pre- and post-immunotherapy. We aim to quantify how therapeutic interventions physically reshape the immune landscape over time. Finally, because our tensors explicitly map the spatial interactions of amino acid pairs, they present a novel pathway toward generative immunology. By defining the spatial probability matrix required for specific antigen binding, future models could invert this tensor space to rationally design synthetic, high-affinity TCR sequences for targeted adoptive cell therapies.
