# Supplementary material for: HELP-TCR: Harmonized Explainable Language Processing Toolkit for T Cell Antigen Receptor Repertoires
Source: Comput Struct Biotechnol J. 2026 Aug 21;35(1):0117. doi: 10.34133/csbj.0117 (PMC13505571; doi:10.34133/csbj.0117)
Supplement: Supplementary file 1 [file csbj.0117.f1.zip › FigS3.pdf]

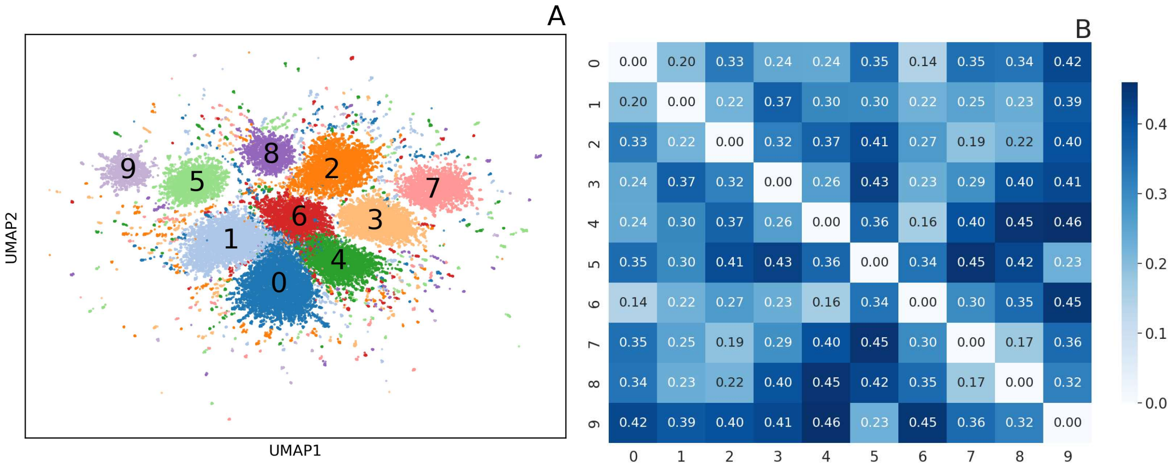
A
UMAP1
UMAP2
9
5
8
2
7
6
3
1
4
0
B
0.00 0.20 0.33 0.24 0.24 0.35 0.14 0.35 0.34 0.42
0.20 0.00 0.22 0.37 0.30 0.30 0.22 0.25 0.23 0.39
0.33 0.22 0.00 0.32 0.37 0.41 0.27 0.19 0.22 0.40
0.24 0.37 0.32 0.00 0.26 0.43 0.23 0.29 0.40 0.41
0.24 0.30 0.37 0.26 0.00 0.36 0.16 0.40 0.45 0.46
0.35 0.30 0.41 0.43 0.36 0.00 0.34 0.45 0.42 0.23
0.14 0.22 0.27 0.23 0.16 0.34 0.00 0.30 0.35 0.45
0.35 0.25 0.19 0.29 0.40 0.45 0.30 0.00 0.17 0.36
0.34 0.23 0.22 0.40 0.45 0.42 0.35 0.17 0.00 0.32
0.42 0.39 0.40 0.41 0.46 0.23 0.45 0.36 0.32 0.00
0 1 2 3 4 5 6 7 8 9
0.0
0.1
0.2
0.3
0.4
