# Supplementary material for: HELP-TCR: Harmonized Explainable Language Processing Toolkit for T Cell Antigen Receptor Repertoires
Source: Comput Struct Biotechnol J. 2026 Aug 21;35(1):0117. doi: 10.34133/csbj.0117 (PMC13505571; doi:10.34133/csbj.0117)
Supplement: Supplementary file 1 [file csbj.0117.f1.zip › FigS4.pdf]

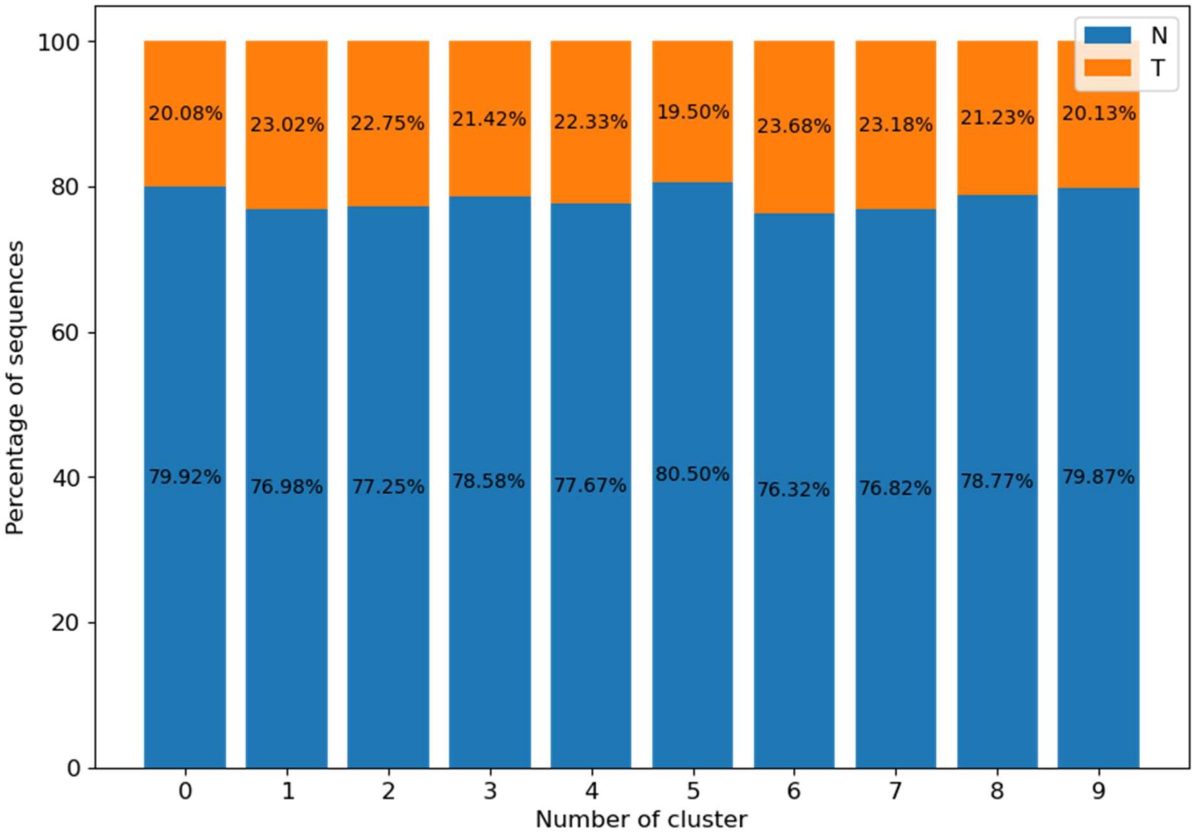
N
T
20.08%
23.02%
22.75%
21.42%
22.33%
19.50%
23.68%
23.18%
21.23%
20.13%
79.92%
76.98%
77.25%
78.58%
77.67%
80.50%
76.32%
76.82%
78.77%
79.87%
Percentage of sequences
0
20
40
60
80
100
0
1
2
3
4
5
6
7
8
9
Number of cluster
