# Supplementary material for: HELP-TCR: Harmonized Explainable Language Processing Toolkit for T Cell Antigen Receptor Repertoires
Source: Comput Struct Biotechnol J. 2026 Aug 21;35(1):0117. doi: 10.34133/csbj.0117 (PMC13505571; doi:10.34133/csbj.0117)
Supplement: Supplementary file 1 [file csbj.0117.f1.zip › FigS5.pdf]

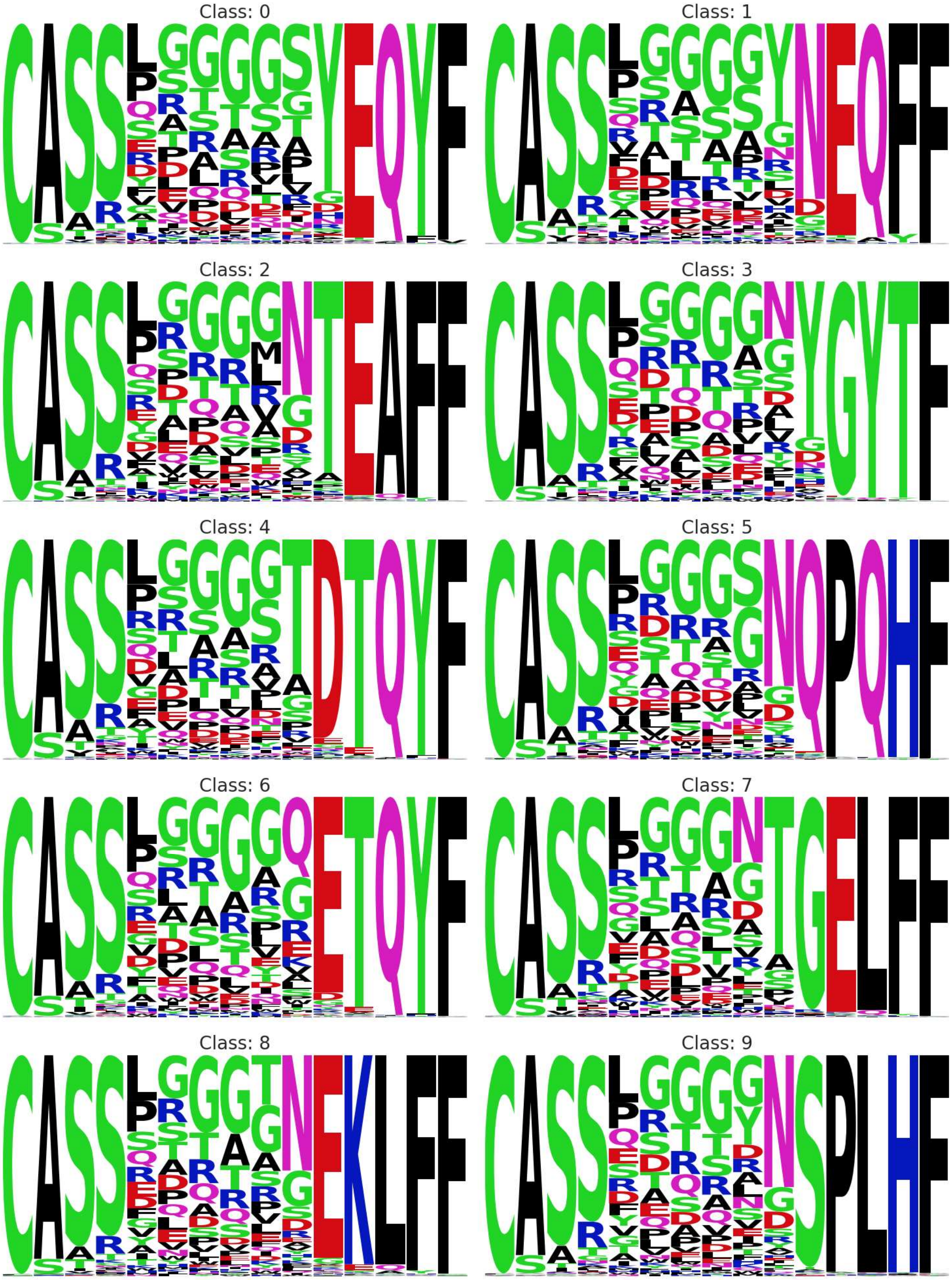
Class: 0
Class: 1
Class: 2
Class: 3
Class: 4
Class: 5
Class: 6
Class: 7
Class: 8
Class: 9
