# Supplementary material for: HELP-TCR: Harmonized Explainable Language Processing Toolkit for T Cell Antigen Receptor Repertoires
Source: Comput Struct Biotechnol J. 2026 Aug 21;35(1):0117. doi: 10.34133/csbj.0117 (PMC13505571; doi:10.34133/csbj.0117)
Supplement: Supplementary file 1 [file csbj.0117.f1.zip › FigS7.pdf]

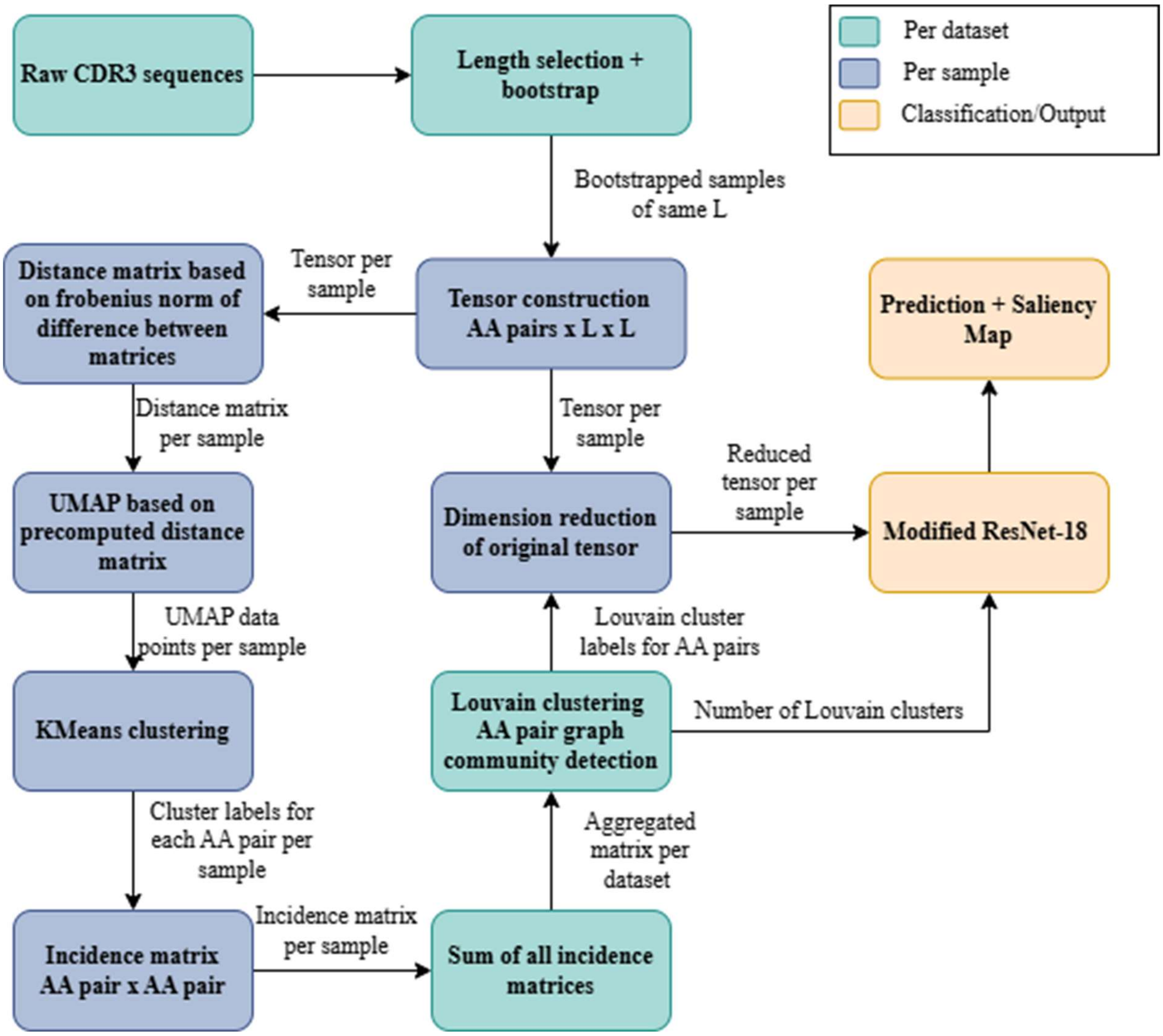
Per dataset
Per sample
Classification/Output
Raw CDR3 sequences
Length selection + bootstrap
Bootstrapped samples of same L
Tensor construction AA pairs x L x L
Tensor per sample
Distance matrix based on frobenius norm of difference between matrices
Distance matrix per sample
UMAP based on precomputed distance matrix
UMAP data points per sample
KMeans clustering
Cluster labels for each AA pair per sample
Incidence matrix AA pair x AA pair
Incidence matrix per sample
Sum of all incidence matrices
Aggregated matrix per dataset
Louvain clustering AA pair graph community detection
Louvain cluster labels for AA pairs
Number of Louvain clusters
Tensor per sample
Dimension reduction of original tensor
Reduced tensor per sample
Modified ResNet-18
Prediction + Saliency Map
