# Supplementary material for: HELP-TCR: Harmonized Explainable Language Processing Toolkit for T Cell Antigen Receptor Repertoires
Source: Comput Struct Biotechnol J. 2026 Aug 21;35(1):0117. doi: 10.34133/csbj.0117 (PMC13505571; doi:10.34133/csbj.0117)
Supplement: Supplementary file 1 [file csbj.0117.f1.zip › List of Supplementary Figures.docx]

# List of Supplementary Figures:

*Figure S1: UMAP visualization of TCR-encoded sequences embedded using TCR-BERT. A two-dimensional UMAP scatter plot representing the learned embeddings of TCR sequences. Each point corresponds to a single TCR sequence. Colors indicate the class to which each sequence belongs (N- normal, T-tumor).*

*Figure S2: UMAP visualization of TCR-encoded sequences embedded using TCR-BERT. A two-dimensional UMAP scatter plot representing the learned embeddings of T-cell receptor (TCR) sequences. Each point corresponds to a single TCR sequence. Colors represent clusters obtained from Leiden clustering applied to the TCR-BERT embedded sequences.*

*Figure S3: A. UMAP-filtered visualization of the first 10 Leiden clusters. Each point represents a TCR sequence, with colors indicating cluster membership. B. Heatmap representing distance matrix of the first 10 clusters, computed using the Wasserstein distance as the similarity measure.*

*Figure S4: A bar plot representing the percentage of sequences in the first 10 clusters. Each bar shows the count within a single cluster. Colors correspond to the class of each sequence sample (N- normal , T-tumor).*

*Figure S5. Logo plots representing characteristic sequences within each cluster chosen for visualization out of Leiden clusters.*

*Figure S6. ROC curve of DeepTCR algorithm applied to our data. The AUC is shown on legend. Y-axis represents a true positive rate. X-axis represents a false positive rate.*

*Figure S7: Schematic overview of HELP-TCR pipeline.*
