# Supplementary material for: HELP-TCR: Harmonized Explainable Language Processing Toolkit for T Cell Antigen Receptor Repertoires
Source: Comput Struct Biotechnol J. 2026 Aug 21;35(1):0117. doi: 10.34133/csbj.0117 (PMC13505571; doi:10.34133/csbj.0117)
Supplement: Supplementary file 1 [file csbj.0117.f1.zip › Supp1.pdf]

Supplement 1: Detailed analyses of the results of TCR BERT and Deep TCR

TCR BERT based classification

The TCR BERT implementation provided cluster information derived from the Leiden algorithm. As an initial step, we examined the scatter plot of the embeddings to assess the distribution and separation of clusters.

The application of the Leiden clustering algorithm to TCR BERT embeddings does not reveal well-defined clusters that naturally represent the data under analysis as shown in Figure S1. Consequently, we focus our analysis on the first 10 clusters, which were sufficiently large to be quantitatively analyzed. For visualization purposes, we provide a filtered scatter plot highlighting the Leiden clusters selected for closer examination. We also provide a distance matrix and as a distance measure we chose Wasserstein distance.

We also compare the counts of sequences belonging to classes N and T. The clustering results do not reveal any inherent classification present in the data, as naturally occurring classes are mixed within the clusters rather than forming distinct, homogeneous groups. This lack of clear separation makes it difficult to further analyze how the algorithm differentiates between samples. Additionally, we provide logo plots for the first 10 clusters to visualize sequence patterns occurring within them.

Deep TCR based classification

As described above, we aimed to assess the variability in the DeepTCR predictions using subsequent random divisions of the data into training and testing. We observed that this approach allowed us to note that the performance of DeepTCR was highly dependent on the dataset split into training, test, and validation sets with testing AUC ranging from 0.47 to 0.99. This is because the provided software takes the percentage of training data as a parameter and automatically divides it into training and validation sets in a 50:50 ratio. To assess the impact of this splitting strategy, we conducted multiple experiments with different data splits. The results were inconsistent, with some cases yielding high accuracy scores on the test set but low AUC values, while others exhibited poor test set performance but high AUC values. To illustrate the substantial influence of dataset splitting on model performance, we provide ROC curves demonstrating these variations.
