# Supplementary material for: HELP-TCR: Harmonized Explainable Language Processing Toolkit for T Cell Antigen Receptor Repertoires
Source: Comput Struct Biotechnol J. 2026 Aug 21;35(1):0117. doi: 10.34133/csbj.0117 (PMC13505571; doi:10.34133/csbj.0117)
Supplement: Supplementary file 1 [file csbj.0117.f1.zip › Supp2.pdf]

Supplement 2: Probabilistic models related to HELP-TCR

Wasserstein distance for comparisons of distributions.

The Wasserstein distance between two discrete probability distributions can be formulated as an optimal transport problem. Set

$$p = (p_i), i = 1, \dots, n \text{ and } q = (q_j), j = 1, \dots, m \text{ where } \sum_{i=1}^{n} p_i = \sum_{j=1}^{m} q_j = 1 \qquad (1)$$

to be two probability distributions over a finite support and let $C \in R^{n \times m}$ be the cost matrix of moving a probability mass between the two distributions. Using the matrix notation, the Wasserstein distance is given by:

$$W(p,q) = \min_{P \in R^{n \times m}} \sum_{i,j} C_{ij} P_{ij}, \text{ subject to } \sum_{j=1}^{m} P_{ij} = p_i \text{ and } \sum_{i=1}^{n} P_{ij} = q_j \text{ for each suitable } i,j \qquad (2)$$

The solution of the problem of minimizing the transport cost is in general not very effective, thus there has been a modification of this problem proposed, which is known as the Sinkhorn regularization. The above defined problem is slightly modified, by adding a penalty for high entropy of the transport matrix. In other words, for each $\epsilon > 0$, we have

$$\begin{aligned} W_{\cdot}(p,q) &= \min_{P \in R^{n \times m}} \sum_{i,j} C_{ij} P_{ij} - \epsilon H(P) \\ &= \min_{P \in R^{n \times m}} \sum_{i,j} C_{ij} P_{ij} + \epsilon \sum_{i,j} P_{ij} \log(P_{ij}) \end{aligned} \qquad (3)$$

This entropy term ensures strict convexity and makes the problem solvable via iterative Bregman projections. In short, one may prove that the regularized transport plan has the following explicit form: $P_{\cdot} = diag(a)\, K\, diag(b)$, where a and b are scaling factors that ensure that the marginal conditions in the definition of the Wasserstein distance are fulfilled. Moreover, we set $K = e^{-C/\epsilon}$ for some $\epsilon > 0$. To compute the scaling factors one uses Sinkhorn iterations, where for a given $t > 1$

$$u^{(t+1)} = \frac{p}{Kv^{(t)}} \text{ and } v^{(t+1)} = \frac{q}{K^T u^{(t+1)}}. \quad (4)$$

It was shown that this procedure converges to the solution of the optimal problem defined by the regularized Wasserstein distance.

We propose to look at this regularization from a different standpoint than the optimal transport problem. The penalty function increases as the transport matrix becomes sparse. Here we also need to consider practical applications when sampling from highly diverse repertoires of TCRs. We arrive at the conclusion that the resulting empirical distributions will have small sample coverage and in extreme cases be dispersed into unit masses. We recall that for sample $\hat{X}$ of size n from the multinomial distribution with categories $K_1, \ldots, K_m$ the sample coverage may be defined as $S := \Sigma_{i=1}^{m} \triangle (K_i \in S)$ where $\triangle (K_i \in S)$ means that category K_i was covered by the sample S. The celebrated Good-Turing estimator of the sample coverage is given by $\hat{S} = 1 - \frac{f_1}{n}$ where f_1 is the number of categories which were represented only once in the sample S.

In this setting, we propose, and postpone all necessary proofs of convergence to a separate manuscript, to use a regularization method based on the Renyi entropy functions with the sample coverage incorporated. In other words we define a Renyi entropy regularized Wasserstein metric as

$$\begin{aligned} W_r(p,q) &= \min_{P \in R^{n \times m}} \sum_{i,j} C_{ij} P_{ij} - \epsilon H_S(P) \\ &= \min_{P \in R^{n \times m}} \sum_{i,j} C_{ij} P_{ij} - \epsilon \frac{i}{i-c} \log(\sum_{i,j} P_{ij}^{c}) \end{aligned} \quad (5)$$

Intuitively, we increase, by the properties of the Renyi entropy (monotonicity in C), the penalty as compared with the Sinkhorn regularization in cases when the repertoire is poorly covered by the sample.

Modeling the TCR repertoire

We start with background definitions. We set

$$A \coloneqq \{A, C, D, E, F, G, H, I, K, L, M, N, P, Q, R, S, T, V, W, Y\} \tag{6}$$

to be the alphabet of available amino acids which are the building blocks of the TCR receptor. Like in many other aspects of language modeling, we focus on the representation of a population of receptors by means of descriptors built on the above alphabet. Below, we present a natural stepwise procedure in which we make our approach more and more complex so it is able to capture subtle differences between the compared repertoires. In the first step, we only use a bag of letters, or a bag of k-tuples (motifs of subsequent amino acids in the TCR beta chain). In this way, given the set of features, which one wishes to analyze - that is for a selected k in the definition of k-tuples - we define $X = \{X_i, i = 1, \ldots, 20^k\}$ to be the finite set of all possible k-tons which will serve as the support of the probability distributions to be compared. Here, we find that it is pointless to bring up to the reader the obvious curse of dimensionality when considering more and more complex sets of features.

For a given X, we define

$$T \coloneqq \{p_i, i = 1, \ldots, 20^k, \sum_{i=1}^{20^k} p_i = 1\} \tag{7}$$

to be the family of all probability distributions over the set X. This is our first representation of the TCR repertoire. In what follows, we propose a set of methods for comparing the elements of T.

For clustering and classification purposes, we allow the user to define a custom distance matrix. Yet we also have a defined family of known similarity measures, like: Minkowski's and Canberra distances, Kullback-Leibler divergence, Renyi's divergence and others. For low numbers of features, that is when k is not large, we recommend using the Wasserstein distance, which we discussed above. For more complex motifs $k > 3$, we do not recommend using this distance, as the distributions tend to be sparse, hence the transport matrices are heavily dependent on the sampling scheme and biased by differences in depth of sequencing and the number of TCR templates - which may be a consequence of differences in diversity of the TCR repertoires.

Example.

In the presented model, for k large enough we fall into the regimen of rare events which do not follow the asymptotics of the Poisson limit theorem (i.e. when $n \cdot p_i \to \lambda$) for some non-zero λ. Consider a multinomial random vector $(X_1, \ldots, X_m)$ where $m = 20^k$ with corresponding probabilities for each category $(p_1, \ldots, p_m)$. To approximate the situation when the sampling coverage is extremely low we consider a series of such distributions where the probability vector depends on n that is $(p_1(n), \ldots, p_m(n))$. We make an assumption that violates the traditional regimen for Poisson approximation by allowing for $\lim_{n\to\infty} n \cdot p_i(n) = 0$ for each i. In such case, for any sample taken from this distribution, the expected number of singletons (sum of categories observed once in the sample) tends to infinity - is elementary to check that this expected number of singletons equals $\Sigma_{i=1}^{m} \frac{n}{1} \cdot p_i \cdot (1 - p_i)^{n-1}$ which tends to infinity with n. Our first conclusion here is that the calculation of Wasserstein matrices in this case in principle limits to computing the number of unit masses to be moved between distributions. To see this consider two independent samples from the same distribution we may calculate the probability that in these two samples a single category is covered in both samples, which (for any i) equals to

$$P(X_i > 0) \cdot P(X_i > 0) = \big(1 - P(X_i = 0)\big)\big(1 - P(X_i = 0)\big) = \\ = \left(1 - \frac{n}{0} p_i{}^0 (1 - p_i)^n\right)^2 \tag{8}$$

and the latter tends to zero as n tends to infinity.

As to the sampling errors and diversity of the TCR repertoire, by the above simple example, we see that with low coverage, one does not expect the available classical tools for comparing the TCR repertoires will provide anything above pseudo-qualitative comparisons. We find that this may be seen as a hint how not to choose the k to be sufficiently high - as low sample coverage may lead to little overlap between two samples drawn independently from the same distribution. We also encourage the users to evaluate both the sample coverage and the diversity of the set X. It may seem to be a classical and a very much simplistic exercise, yet we claim that in certain cases the two estimators may lead to interesting interpretation. For the sample coverage we have seen that in certain cases we may use it to avoid the curse of dimensionality. Yet, we have to remember that we are analyzing a specific set of peptides, which have both the 3 prime and the 5 prime end much less diverse than the central positions. These 3 prime and 5 prime ends

contain germline encoded sequences selected from a finite pool of possible segments. Having this in mind, we also propose to compare the diversity of the distributions of the k-tuples as long conserved motifs corresponding to the germline encoded segments of the receptor introduce a type of mixture distribution over the set of motifs and the analysis of this mixture (for instance via the Jensen-Renyi divergence) may inform us on the gene usage bias without the cumbersome combinatorial approach to estimating the exact abundance of each germline segment.
