# Supplementary material for: HELP-TCR: Harmonized Explainable Language Processing Toolkit for T Cell Antigen Receptor Repertoires
Source: Comput Struct Biotechnol J. 2026 Aug 21;35(1):0117. doi: 10.34133/csbj.0117 (PMC13505571; doi:10.34133/csbj.0117)
Supplement: Supplementary file 1 [file csbj.0117.f1.zip › Supp3.pdf]

Supplement 3: Implementation of HELP-TCR in Python

The library contains three modules for TCR data analysis:

- Logo plots plotting module
- EMD (Wasserstein metric) matrix clustering
- Deep Learning module with ResNet.

We implemented the *DataLoader* abstract class which is an universal data loader and works as a generator loading data in a lazy way for saving operating memory. It has the virtual method *_transform_data_* which has to be implemented in child classes and works as a data transformer to adapt data for further receivers. There is a series of children DataLoader classes:

- Bootstrap – for data normalization
- EMDLoader – for calculating probability of amino acids on particular position in peptide
- ProbLoader – calculating probability of occurrence of all pairs of aminoacids on particular positions
- FrobeniusLoader – calculating Frobenius norm on probability tensors
- IncidenceLoader – performing UMAP and KMeans and estimates incidence matrix of a aminoacids pairs in common clusters
- Mapper – it maps each sample to a class label calculated with a Louvain algorithm basing on incidence matrix from above class.

All classes are generators and returns a stream of data between each other.

There is also a Reducer class that behaves as a function and it clusterizes all samples by incidence matrices from IncidenceLoader and returns a list of labels defining a communities in data and *LoadTCRData* class that prepares data as a streams of tensors for deep learning methods divided on training data, testing data and validation data.

The library implements the *EMDProcessor* class that behaves as a function. It receives the *EMDLoader* object and returns the EMD distance matrix.

The library implements the *LogoPlot* class that behaves as a function. It receives the *LogoLoader* object and returns logoplot figure.

The library implements the *DeepLearningTCR* class. It receives the *LoadTCRData* object and allows for the following methods:

- train() for ANN (Artificial Neural Network) training
- evaluate() for estimating ANN efficiency
- predict() for class prediction of a new sample

Additionally, there is a *BackupLoader* class that is working as a backuper. In case of emergency it is able divide streams to multiple destinations and save stream on a hard disc. We also implemented a *TCRManager* class that operates on the classes above and delivers an interface to use for users. The library is available via https://zenodo.org/records/17944547

Model parameters used for training:

The manuscript shows cut-off = 0.238 on the ROC curve (Fig. 11E/12A). This was chosen by maximizing Youden's J statistic on the validation set. We used Stochastic Gradient Descent (SGD) with momentum 0.05 and a fixed learning rate of 0.0001, trained for 30 epochs with a batch size of 16. No weight decay or early stopping was applied. Class imbalance was addressed via a weighted cross-entropy loss with per-class weights [1.0, 2.0] assigned to the majority and minority class, respectively, combined with label smoothing of 0.1 to reduce overconfidence. Random seeds were fixed across NumPy, PyTorch, and Python's random module to ensure reproducibility.
